# Intelligent Systems for Space Conditioning and Shading in Buildings: A Systematic Review of Artificial Intelligence Techniques and Their Energy Impact

**DOI:** 10.3390/s26206405

**Published:** 2026-10-10

**Authors:** Tomás Uribe-Vargas, Aner Martinez-Soto, Daniel Sanhueza-Catalán, Millaray Curilem-Saldías, Rafael Batres

**Affiliations:** 1 Department of Civil Engineering, Faculty of Engineering and Sciences, Universidad de La Frontera, Temuco 4811230, Chile; tomas.uribe@ufrontera.cl (T.U.-V.); dsanhuezacatalan@gmail.com (D.S.-C.); millaray.curilem@ufrontera.cl (M.C.-S.); 2 School of Engineering and Sciences, Tecnológico de Monterrey, Monterrey 64849, Mexico; rafael.batres@tec.mx

**Keywords:** artificial intelligence, thermal comfort, energy efficiency, dynamic shading, HVAC control, building automation, systematic review, sensors, occupancy detection

## Abstract

**Highlights:**

**What are the main findings?**

- AI applied to HVAC shows greater experimental maturity than solar management.
- Real-world validation reaches 31% of HVAC studies but only 14% of solar-management studies.

**What are the implications of the main findings?**

- Predictive and data-driven control validated in HVAC is a promising, still largely untested, route toward adaptive solar-shading control.
- Closing this gap requires long-term real-world validation in underrepresented climates, with comparable baselines and the sensing of anticipatory variables.

**Abstract:**

Artificial intelligence (AI) is increasingly applied to heating, ventilation, and air-conditioning (HVAC) control, whereas solar-radiation management still relies mainly on reactive strategies; whether AI capabilities validated in HVAC could be transferred to solar control has not been systematically assessed. This review compares both domains in terms of experimental maturity, climatic coverage, and input variables. Web of Science studies (2015–2026) reporting quantitative energy-demand variations were systematically reviewed following PRISMA 2020 and synthesized descriptively, with a sensitivity search adding reinforcement-learning and predictive-control terms. Fifty-six studies were included (29 HVAC, 28 solar, 1 shared). HVAC reductions ranged from 3.8% to 62.4% (median 22.1%), a spread associated more with heterogeneous baselines, durations, and metrics than with techniques. Real-world studies represented 31% in HVAC versus 14% in solar management, where AI-based predictive control appeared in one real-world study. Anticipatory variables were scarce but coincided with some of the highest savings, a non-causal association. Model-level patterns, such as Random Forest replication, were not preserved when 18 additional studies were considered. Transferring predictive control from HVAC to solar management is a promising line that requires long-term real-world validation in underrepresented climates. Limitations include a single database and single-reviewer screening. The review was not registered or externally funded.

## 1. Introduction

It is projected that, without improvements in the energy efficiency of buildings, the rise in urban temperatures associated with climate change and heat islands could triple the energy demand for cooling by 2050 [1,2]. This phenomenon not only increases the energy demand but also aggravates cardiorespiratory diseases, especially in vulnerable populations; for this reason, several authors emphasize the urgency of reducing the energy demand without compromising the habitability and well-being of the occupants [3,4,5]. In this context, several authors have begun to develop solutions based on artificial intelligence (AI) to manage the energy operation of buildings and to reduce the energy demand without affecting the hygrothermal comfort (HTC) conditions [6,7,8,9,10]. These solutions rely on the prediction of environmental variables, the optimization of energy consumption, or real-time adaptive control [11,12,13,14,15]. Simple adaptive rule-based systems grounded in fuzzy logic achieve reductions of close to 32%, demonstrating the capacity to manage heating, ventilation, and air-conditioning (HVAC) operating strategies [16,17,18,19]. Machine-learning techniques such as Random Forest have managed to reduce the energy demand by up to 43% through the prediction of both environmental variables and variables related to occupancy patterns [20,21]. In addition, intelligent control approaches such as Model Predictive Control (MPC) have made it possible to anticipate the thermal demand and manage HVAC systems under dynamic scenarios, achieving energy-demand reductions of up to 63% [22,23,24,25]. These functions not only reduce the energy demand but also improve the HTC and lighting conditions, especially in dynamic, high-occupancy contexts such as offices [26]. Nevertheless, most of these applications have been oriented toward counteracting the effects of the climatic environment through control of the HVAC system, without exploiting the potential of strategies that would make it possible to manage or even harness exterior conditions such as solar radiation [27].

Solar radiation is an indispensable variable for reducing the energy demand and improving the HTC in buildings [28]. In this context, technologies such as electrochromic (EC) windows and Suspended Particle Devices (SPDs) have been developed, which make it possible to limit the incident radiation and lower the energy demand of buildings, achieving reductions of up to 54% in hot climates and 56% in temperate climates [29,30,31]. Nevertheless, their performance depends on the control strategy; comparative studies between manual and automated control have shown energy-demand reductions of between 10% and 30% [32,33]. Various studies have shown that solar-radiation management systems, using simple rules or thresholds, make it possible to achieve a better balance among HTC, visual comfort, and energy efficiency, in both simulations and real-world applications [4,34].

Both domains act on the same thermal balance. Solar gains through the envelope are one of the main loads that HVAC systems must compensate, so the operation of shading devices directly modifies the heating and cooling demand, and vice versa. Integrated control studies have shown that coordinating air-conditioning, lighting, and shading reduces electricity consumption by 15–21% compared with conventional control [35]. Reviewing both domains together therefore makes it possible to identify which capabilities already validated in HVAC could be transferred to solar control and which limitations are shared by both.

Despite all the advances achieved by AI algorithms applied to HVAC systems, these have not yet been transferred to the field of solar-radiation management, where strategies remain static and lacking any adaptive capacity [36]. These systems remain basic, dominated by thresholds and simple rules [6,37,38,39,40]. Recent reviews confirm that the machine-learning control of dynamic façades is still at an early stage compared with rule-based and model-based methods [41], although the AI-enabled predictive control of kinetic façades has begun to be demonstrated [42]. This gap—the absence of a systematic transfer of the AI capabilities already validated in HVAC to solar control—is the starting point of this study. In this context, several authors point to the need for intelligent and autonomous systems, highlighting this gap as the next evolutionary stage in the control of automated façades [34,43]. Moreover, the authors point to the need not only for more research but also for diversifying studies across different types of climates, encompassing tropical, arid, and humid regions [43,44,45]. Likewise, a limited exploration of the selection and combination of input variables is evident, in which easily accessible signals predominate, even though contextual, occupancy-related, and predictive variables have been associated with larger reductions in energy demand [46,47,48]. Even in HVAC, experimentally validated adaptive control remains limited: only about a quarter of reinforcement-learning studies have been deployed in real buildings [49,50].

To address this gap, and unlike previous reviews that examine AI for HVAC or dynamic façades separately, this review compares both domains within a common framework of experimental maturity, climatic coverage, and input variables, including the sensing technologies that provide them. Specifically, this research aims, first, to identify the AI approaches and algorithms most widely used in HVAC systems, in order to characterize the methodological and operational patterns that predominate in the literature. Second, it seeks to describe the characteristics reported for solar-radiation management systems, considering their level of automation, the types of strategies implemented, and the stage of development reached. Third, the study proposes to analyze the climatic distribution of the existing research, with the aim of recognizing trends and gaps in the application of AI in tropical, arid, and humid climates. Finally, it seeks to examine the input variables used in the reviewed models, identifying their frequency and function within the control schemes, with the aim of providing guidelines for future developments oriented toward integrated energy-management systems in buildings. Because the input variables of these controllers are provided by physical sensors (temperature, illuminance, occupancy, or biometric sensors) or by virtual sensors (weather and production forecasts), this last objective also characterizes the sensor-to-control integration on which the transfer between domains depends [51,52,53].

## 2. Materials and Methods

Based on the niches identified in the literature, the study set out to answer four research questions aimed at characterizing the trends reported in the application of AI to HVAC systems and to solar-radiation management technologies. These questions address the observed level of automation, the way in which climate conditions the available research, the selection and use of input variables in control models, and the characteristics described for the operation of the systems. The purpose is to offer a comparative overview in terms of technological maturity and methodological patterns, without interpreting the reported values as comparable metrics or as direct indicators of energy performance.

- Which artificial intelligence approaches and algorithms are reported most frequently in the literature applied to HVAC systems, and what functions do they perform?
- What is the level of automation and complexity of current solar-radiation management systems, and to what extent do they integrate principles of intelligent or autonomous control?
- How are AI and solar-radiation management applications distributed across the different climatic groups, and what patterns or gaps are observed in terms of climatic coverage?
- Which input variables are incorporated most frequently in AI models, and how do they contribute to structuring the prediction and control strategies described in the literature?

To answer these questions, a systematic literature review was conducted with the aim of characterizing the current state of the application of AI techniques in HVAC systems and in solar-radiation management technologies in buildings. The methodology followed the guidelines established by the Preferred Reporting Items for Systematic Reviews and Meta-Analyses (PRISMA) 2020 statement [54], structuring the analysis around two main axes: (1) the use of AI algorithms in HVAC systems and (2) the control strategies and level of automation present in dynamic solar-radiation management technologies.

Figure 1 depicts the workflow of both search axes for the selection of studies and the descriptive analysis of the development of both technologies.

### 2.1. Systematic Literature Review According to PRISMA

This systematic review is reported in accordance with the PRISMA 2020 statement. The completed PRISMA 2020 checklist [55] is provided in the Supplementary Materials. The review was not prospectively registered, and no formal review protocol was prepared.

The systematic review was organized around two thematic axes: (1) AI techniques applied to HVAC systems and (2) solar-radiation management technologies. The search was carried out in Web of Science (WoS), using combinations of terms related to AI, hygrothermal comfort, and HVAC systems in the first axis, and to dynamic shading, solar control, and variable-transmission windows in the second. The search period was limited to between 2015 and 2026. The year 2015 was set as the starting point because it coincides with the consolidation of deep learning and deep reinforcement learning as control tools, following the demonstration of deep Q-networks [56]; in addition, earlier output at this intersection is marginal (fewer than five records in 2015), and automatic control strategies for façades and HVAC systems prior to this period have been covered by previous reviews [36,57]. The searches were run on 20 January 2026.

- Axis 1 (AI applied to HVAC)—query executed in the Web of Science Core Collection: TS = ((“artificial intelligence” OR “machine learning” OR “deep learning” OR “neural network*” OR “LSTM” OR “fuzzy logic”) AND (“thermal comfort” OR “hygrothermal comfort” OR “indoor environment*”) AND (“HVAC” OR “heating ventilation and air conditioning” OR “air conditioning” OR “space conditioning”) AND (“residential building*” OR “housing” OR “office building*” OR “workplace*”)).
- Axis 2 (solar-radiation management)—query executed in the Web of Science Core Collection: TS = ((“dynamic shading” OR “solar shading” OR “solar control” OR “automated blind*” OR “smart window*” OR “dynamic glazing” OR “intelligent shading system*”) AND (“thermal comfort” OR “hygrothermal comfort” OR “indoor environment*”) AND (“residential building*” OR “housing” OR “office building*” OR “workplace*”)).

The term blocks were structured in three dimensions (technique or device, comfort or indoor environment, and building type), following the structure used in related reviews [6,36,44]. Façade-morphology terms such as kinetic façade were not included in Axis 2 because the object of analysis is the control strategy of the solar-management device rather than the geometry of the envelope; kinetic-façade studies that evaluate the control of shading elements are captured by the terms dynamic shading, solar shading, and solar control.

During the revision of the manuscript, a sensitivity search for Axis 1 was run in the Web of Science Core Collection (16 September 2026), explicitly adding the terms reinforcement learning, deep reinforcement learning, and model predictive control: TS = ((“artificial intelligence” OR “machine learning” OR “deep learning” OR “reinforcement learning” OR “neural network*” OR “LSTM” OR “fuzzy logic” OR “deep reinforcement learning” OR “model predictive control”) AND (“thermal comfort” OR “hygrothermal comfort” OR “indoor environment*”) AND (“HVAC” OR “heating ventilation and air conditioning” OR “air conditioning” OR “space conditioning”) AND (“residential building*” OR “housing” OR “office building*” OR “workplace*”)). The search retrieved 170 records, of which 76 had not been assessed previously and were screened against the same eligibility criteria; 18 additional studies met the criteria (Figure S1, Table S1). These studies were not integrated into the main analysis but were used to assess the robustness of its findings (Section 3.5). They were kept separate because the expanded search was defined during peer review; merging this post hoc search with the original one would have altered the selection process reported in Figure 2.

The Web of Science Core Collection was used as the sole source for three reasons. First, it indexes only journals that pass an editorial evaluation process, which is consistent with the criterion of including exclusively peer-reviewed studies with quantitative results. Second, it supports reproducible field-based Boolean searches, a requirement that non-curated sources such as Google Scholar do not meet for systematic reviews [58]. Third, most journals indexed in Web of Science are also indexed in Scopus; although Scopus offers broader coverage, the core peer-reviewed journals of engineering and the applied sciences are shared by both databases [59]. Grey literature was excluded because the eligibility criteria require peer-reviewed quantitative results. The use of a single database is acknowledged as a limitation (Section 4).

Inclusion criteria were defined by axis. Axis 1 included studies that applied AI techniques (including model predictive control and reinforcement learning, following the taxonomy in [57]) to the control of HVAC systems. Axis 2 included studies that evaluated automated control strategies for dynamic solar-radiation management devices at any level of automation, from threshold and rule-based control to optimization and predictive control; this broader criterion reflects that research question ii aims precisely to characterize the level of automation of these systems, not only AI-based cases. In both axes, studies had to be original peer-reviewed articles addressing residential buildings or offices, under real or simulated conditions, and reporting quantitative variations in space-conditioning energy demand (heating, cooling, or total system demand). Theoretical reviews, predictive studies without validation, passive approaches without automation, and works lacking a complete methodology or quantifiable results were excluded. Hereafter, the term AI–shading intersection is reserved for Axis 2 studies that use predictive or data-driven control.

Selection proceeded in two stages: (i) screening of titles and abstracts and (ii) full-text assessment applying the inclusion and exclusion criteria. Records from each axis were exported from Web of Science in Excel format, and the single duplicate between axes was identified manually by comparing titles and DOIs. Title/abstract screening and full-text assessment were conducted manually in a spreadsheet, recording for each record the inclusion decision and the reason for exclusion. For each accepted study, general data, the AI technique or type of solar-management system, the input variables used, and the energy variations reported by each study were recorded. The complete process is summarized in Figure 2 (PRISMA).

Study selection was primarily performed by T.U.-V. A second reviewer, D.S.-C., independently screened a randomly selected sample of 35 title-and-abstract records from the solar-radiation management axis and reassessed all studies in the final included set against the eligibility criteria. All remaining screening and full-text eligibility decisions were made by T.U.-V. Disagreements were resolved by consensus. The individual decisions for that subsample were not documented record by record; therefore, an inter-reviewer agreement statistic cannot be calculated retrospectively. Records from the sensitivity search were screened and assessed by T.U.-V. No automation tools were used in the selection process.

Data were extracted from each included study by T.U.-V. using a structured data-extraction form. No independent duplicate data extraction was performed, and no automation tools were used. When a study reported eligible results for different climates or experimental configurations, all relevant estimates were extracted. Multiple estimates from the same study were treated as non-independent observations, and each study was counted only once in the total number of studies.

From the selected studies, the temporal and geographical characterization of both lines of research was developed, identifying the year and country of publication in order to analyze the evolution of work on AI applied to HVAC and on solar-radiation management technologies.

The principal extracted outcome was the percentage change in energy demand reported by each study relative to its own comparator. Because the definition of energy demand, comparison baseline, evaluation period, and experimental conditions varied substantially, these percentages were not assumed to represent a directly comparable common effect measure and were synthesized descriptively.

No standardized risk-of-bias instrument was applied, because the included works span heterogeneous designs—real-world experiments, full-year monitoring campaigns, and short simulations—for which no single validated tool provides comparable domains. Instead, the methodological characteristics most likely to bias the reported energy savings were used as structural information in the synthesis. For every study, the setting (real-world versus simulation), the evaluation duration (from a few hours to two years), and the definition of the energy outcome (heating, cooling, or total HVAC demand) were extracted, together with code availability for Axis 1; these characteristics are systematized in a structured appraisal table (Appendix C). The type of comparison baseline (conventional or rule-based control, manual control, a static device, or another algorithm) and the clarity of attribution (whether the effect of the control was isolated or bundled with parallel measures) were not coded systematically for all studies; they are discussed in Section 3.1, Section 3.2, Section 3.3 and Section 3.4 for the studies in which they condition the interpretation of the reported values, so that results obtained under weak baselines, short horizons, or bundled attribution are flagged rather than treated as equivalent to those from more robust designs. This structured appraisal does not constitute a formal, scored risk-of-bias assessment, and both its partial coverage and the absence of such an assessment are acknowledged as limitations (Section 4).

Given the substantial heterogeneity in comparison baselines, evaluation horizons, outcome definitions, and reported metrics, no quantitative meta-analysis was performed. Results were synthesized descriptively, and the reporting of the synthesis was informed by the Synthesis Without Meta-analysis (SWiM) guideline. Results were organized by functional category, specific AI model, type of solar-radiation control, Köppen climate, and input variable. Individual study estimates were presented in tables and comparative plots, while, for each grouping, the median and interquartile range (IQR; quartiles by linear interpolation) were calculated over all reported values (cases), so that a study evaluating several climates or configurations contributes one case per reported value; the number of independent studies (*n*) and, where relevant, the number of cases are reported, medians are shown only for groupings with five or more cases, and, for smaller groupings, the individual values are reported. No tests of statistical significance were applied; differences between categories are described as patterns observed in the available sample and not as statistically established performance differences. No pooled summary estimate was calculated. Values referring to heating, cooling, or total demand were retained as reported by each study and are identified in Appendix A and Appendix B; in Axis 1, the groupings combine these outcome types and are therefore read descriptively, whereas, in Axis 2, the medians by input variable are calculated on total savings only. Throughout the text, reductions in energy demand are reported as positive percentages and increases as negative values; in the figures of the Section 3, following the convention of the extraction database, reductions are plotted as negative values and increases as positive values. The Axis 2 figures plot total HVAC demand values (for the studies based on seasonal campaigns, the variation measured over the evaluated season), so the two Axis 2 studies that report only heating or cooling values are listed in Appendix B but not plotted. A sensitivity analysis based on the expanded Axis 1 search described above was performed (Section 3.5).

No formal assessment of reporting bias was conducted. Funnel plots and statistical tests for funnel-plot asymmetry were not considered appropriate because comparable effect estimates and measures of precision were unavailable. Potential publication and selective-reporting biases were therefore acknowledged as limitations rather than formally assessed. No formal framework was applied to assess the certainty of the body of evidence; consequently, the conclusions are presented as descriptive patterns in the available literature rather than as estimates supported by a formally rated level of certainty.

### 2.2. Analysis of Studies on AI Applied to HVAC Systems

The analysis of studies on AI applied to HVAC systems was structured at three levels: (1) the operational function of the algorithm (prediction, optimization, control, or hybrid combinations); (2) the AI technique employed (Machine Learning, Artificial Neural Networks, Nature-Inspired Intelligence, Fuzzy Logic, and Model Predictive Control); and (3) the specific model implemented in each study. To organize the identified techniques, the methodological taxonomy proposed by Antonopoulos et al. (2020), which groups the approaches applied in intelligent buildings according to their computational basis and operational function, was adopted [57]. A study was classified as prediction when the model estimates future variables (temperature, demand, and occupancy) without directly intervening in system control; as optimization when it adjusts operating parameters according to a declared objective function; as control when the model directly generates the actuation signals of the HVAC system; and as hybrid when the scheme combines two or more of these functions in an integrated manner.

For each study, the main declared technique, the input variables used, and the reported energy variations were recorded. With this information, a descriptive analysis of the patterns observed in each functional category, AI technique, and specific model was developed.

The climatic analysis was carried out in two stages. In the first, the energy variations reported for each functional category were related to the Köppen climatic groups corresponding to the studied cases, in order to represent descriptively the values reported for each climate. In the second, this analysis was disaggregated by AI model applied in each climate type, which made it possible to visualize the values reported for each model–climate combination and to recognize application patterns and gaps in certain climatic contexts.

Finally, the input variables used in the training or operation stages of the models were identified. Their frequency of occurrence was quantified and their reported role within the analyzed control schemes was characterized, in order to describe their presence in the literature and their relationship to the different methodological approaches observed.

### 2.3. Analysis of Studies Applied to Solar-Radiation Management Systems

The analysis of studies on solar-radiation management was organized around two classification criteria: (1) the type of technology evaluated, including automated blinds, movable awnings, windows with variable optical control, and systems based on the modulation of the solar heat gain coefficient, and (2) the type of control implemented, considering fixed thresholds, seasonal control, automatic multivariable control, and optimization strategies based on genetic algorithms or on simulation procedures. On the basis of these categories, the energy variations reported in each study were described, emphasizing their distribution and range without establishing direct comparisons between technologies. Each Axis 2 study was assigned to a control category according to the decision mechanism declared by its authors: threshold control (TC) when actuation depends on a fixed limit value of a single variable; seasonal control (SC) when the actuation criterion changes with the time of year; rule-based automatic control (RBAC) when several predefined logical rules are combined; and automatic control algorithm (ACA) when a predefined, non-adaptive control sequence is applied. Multivariable automatic control (MVAC) was assigned when more than one input signal is integrated in real time without explicit optimization. Strategies with an explicit search for the optimal state were separated according to the procedure used: simulation-optimized automatic control (SOAC), genetic-algorithm optimized control (GA-OPT), or a parametric optimization algorithm (POA). Finally, data-driven predictive control (DMPC-PC) was assigned when decisions are taken over a prediction horizon using data-driven models.

The climatic analysis was based on the Köppen climate types declared in the studies. For each technology–climate combination, the reported energy variations were recorded, in order to represent descriptively the values documented in different climatic contexts and to recognize application patterns or gaps in certain regions.

Subsequently, the input variables used in the control mechanisms were identified and their frequency of occurrence was recorded. The analysis considered thermal, luminous, contextual, and operational variables, such as indoor and outdoor temperature, solar radiation, illuminance, occupancy, energy prices, and renewable generation. Their presence in the models and their declared role within the control strategies were described, without establishing causal relationships with the reported energy variations.

The analysis was structured to answer the four research questions. For question i, the energy reductions reported in the studies that apply AI techniques to HVAC systems were documented, describing their variability and the patterns observed in relation to the functional category, the technique employed, and the model applied. Question ii was addressed through the classification and characterization of the technologies and control types present in solar-management systems. Question iii was analyzed by integrating the Köppen climatic classification into both study axes and describing the application patterns observed in each climatic group. Finally, question iv was addressed by quantifying the frequency and use of input variables in the reviewed models, describing their role within the analyzed control schemes.

## 3. Results and Discussion

The process of identifying and selecting studies followed the PRISMA 2020 statement, considering the two research axes, the application of AI techniques in HVAC systems and solar-radiation management technologies. A total of 195 records were retrieved in Web of Science, distributed across 126 related to AI applied to HVAC and 69 focused on solar-radiation management. After removing one record shared by both axes, 194 unique records remained for screening. After applying the selection criteria, 29 and 28 studies, respectively, were included. A single study (Klanatsky et al., 2025) was identified by both axes, as it integrates an AI scheme for space conditioning that incorporates solar radiation and the operation of exterior blinds within the same control system in a real-world environment [60].

Figure 2 summarizes the search and article-selection flow. Of the 194 records screened, 77 were excluded during the title and abstract screening because they did not apply AI to HVAC control (Axis 1) or automated control to solar-radiation management devices (Axis 2) in buildings. In the full-text assessment, 117 articles were examined in detail; of these, 61 documents were discarded: 24 because they were theoretical studies, 19 because they presented predictive approaches without automation systems, 6 because they lacked experimental validation, and 12 because of methodological insufficiency or the absence of quantified results. Finally, 56 studies were included, all with active control and evidence of variations in energy demand.

The temporal and geographical distribution of the 195 records retrieved across the two search axes between 2015 and 2026 is presented in Figure 3, which captures both the cumulative number of publications by continent and the annual evolution.

The annual output rose from fewer than 5 studies in 2015 to 37 in 2022, with a moderate decline in subsequent years. Asia concentrates 102 records (52% of the total), followed by the Americas with 40 (21%), Europe with 38 (20%), Oceania with 9, and Africa with 6. In Axis 1, Asia accounts for 56% of the work (71 records), the Americas 27%, Europe 20%, Oceania 6%, and Africa 2%. In Axis 2, Asia represents 45% (31 records), Europe 18%, the Americas 13%, Africa 4%, and Oceania 35. The most active countries in solar-radiation management are China, the United States, Italy, and South Korea, which, together, account for more than 45% of the publications.

The observed geographical concentration is related to the availability of funding, experimental infrastructure, and simulation platforms in the economies leading energy research. Asia and the Americas together account for nearly 80% of Axis 1, partly because of the consolidation of energy-efficiency programs in China, Korea, and the United States. Europe maintains a stable and climatically diversified output, whereas Africa, Oceania, and Latin America appear underrepresented.

### 3.1. Artificial Intelligence Approaches and Algorithms in HVAC Systems and Their Operational Functions

The AI techniques applied to HVAC are grouped into functional categories (prediction, optimization, control, and hybrid combinations). It was found that 20 of the 29 studies (69%) are developed in simulation environments, mainly residential buildings, whereas the 9 real-world studies (31%) are concentrated in offices with documented, rule-based operating baselines. Figure 4 shows the energy-variation values reported by functional category.

The hybrid categories (prediction + control and control + optimization) bring together the largest number of cases, driven by the studies incorporated in recent years. The highest values of the set are distributed across categories: the 62.4% reported by Nengroo et al. (2025) in control + optimization, and the 57.4% of Kannan et al. (2020) and the 43.5% of Manjarres et al. (2017) in prediction [11,21,25]. At the opposite extreme, the prediction + control category concentrates most of its values below 15%, with cases such as Pavirani et al. (2024) at 9%, Sha et al. (2025) at 10.2%, and Xiao et al. (2023) at 5.8% [8,61,62].

The heterogeneity of the values is associated with three methodological factors that Figure 4 does not differentiate. First, for the comparison baseline, the 62.4% of Nengroo et al. (2025) is measured against another reinforcement-learning algorithm (deep deterministic policy gradient, DDPG), not against a conventional or rule-based control; and the 57.4% of Kannan et al. (2020) is compared against manual user control [11,25]. Second, for the duration of the experiment, 9 of the 29 studies (31%) have a duration of two weeks or less, whereas 11 studies cover a year or more, and, of these, 4 correspond to real-world implementations (Manjarres et al. 2017, Kannan et al. 2020, Bünning et al. 2022, and Klanatsky et al. 2025) [11,21,24,60]. Third, for the reported metric, some values refer only to heating or cooling demand, whereas others refer to total HVAC demand (Appendix A).

The dispersion observed between functional categories coincides with these configuration differences rather than with the operational properties of each category. The highest values of the set are not comparable with one another or with the lowest ones; they correspond to different baselines and different evaluation horizons. A reproducible characterization of the state of the art requires that future reports specify the type of baseline, the duration of the experiment, and the metric used as structural information, not as optional data.

Figure 5 presents a scatter plot with the energy-variation values reported in the literature for studies applying different artificial intelligence techniques to the operation of HVAC systems. Each point represents an individual case documented in the reviewed studies, making it possible to visualize the distribution and range of the results associated with each technique.

Artificial Neural Networks (ANNs) and Machine Learning concentrate the largest number of cases with wide dispersion: the ANN ranges between 3.95% (Liao et al. 2025) and 57.4% (Kannan 2020), whereas Machine Learning includes values between 7.6% (Jin 2017) and 43.5% (Manjarres 2017) [11,21,23,63]. Fuzzy Logic concentrates values between 3.8% (Yau 2018) and 32.8% (Chaouch 2021), whereas Model Predictive Control ranges between 20.5% (Ławryńczuk 2019) and 41% (Klanatsky 2025) [16,19,22,60].

The heterogeneity within each technique coincides with differences in implementation characteristics. The dispersion of ANNs reflects the diversity of architectures and training schemes; Kannan et al. (2020) employs a DNN trained on two years of real operation, whereas Mousavi et al. (2023) uses a conventional ANN on an annual simulation [11,14]. Both are labeled as ANNs in Figure 5. In MPC and Fuzzy Logic, the bounded values coincide with structured control schemes, although the dispersion persists when data-driven or adaptive variants are introduced. The technique as a category aggregates, under a single label, models that differ in architecture, training set, and functional role within the control scheme.

The level of aggregation by technique is insufficient to identify reproducible patterns. The presence of techniques with a single case (Nature-Inspired Intelligence) alongside others with a high internal dispersion (ANN, ML) prevents the establishment of expected ranges. This limitation justifies disaggregating the analysis to the level of the specific model, where concrete decisions about the architecture, input variables, and functional role can be examined separately.

Table 1 summarizes the AI models identified in the reviewed studies, including their acronyms and a brief description of their computational structure.

In line with this disaggregation, the analysis now focuses on the specific models implemented within each artificial intelligence technique. Figure 6 presents a scatter plot that gathers the energy-variation values reported for each model identified in the literature, where each point corresponds to an individual case. This representation makes it possible to examine the distribution and range of the results at the model level, providing a more detailed framework for analyzing the configurations and design decisions underlying the reported values.

Figure 6 shows that the energy-variation values are concentrated in distinct ranges according to the specific model implemented. Random Forest is evaluated in two independent real-world studies that report close values, 43.5% in Manjarres et al. (2017, RFR, office, 1 year) and 42% in Levinson et al. (2024, RFC, office, 5 months) [20,21]. Generic Deep Reinforcement Learning groups three cases at the low end, 3.95% in Liao et al. (2025), 8% in Lissa et al. (2021), and 9.7% in Kathirgamanathan et al. (2021); the recent variants of this family, by contrast, report medium and high values [7,63,64]. DQN reaches 27.3% in a nine-month real-world implementation (Ma and Liu. 2026) and 31% in an annual simulation (Maroofiazar and Reveshti 2026); PPO records 35.9% (Zhang et al. 2026), and an occupancy-oriented convolutional neural network (CNN), grouped under ANN in Figure 6, records 52.1% (Liu et al. 2025) [65,66,67,68]. MPC appears as the primary model in two studies (7.6% and 20.5%); when all studies that embed a model-based MPC layer are considered, regardless of the model under which they are plotted (the data-driven MPC and ARMAX-based schemes are analyzed as separate models), the group comprises seven studies (*n* = 7) with a median of 12.3% (IQR 8.9–23.0%; range 5.8–30.7%), which indicates considerable dispersion within the same family and reinforces that the type of control alone does not explain the outcome. The remaining models with notable values each correspond to a single study, QRL with 62.4% (Nengroo 2025), DNN with 57.4% (Kannan 2020), MLR with 43% (Abdellatif 2022), DMPC with 41% (Klanatsky 2025), and ARMAX with 35% (Bünning 2022) [11,13,24,25,60].

The highest value of the set, QRL with 62.4% (Nengroo 2025), coincides with two conditions, the baseline is another reinforcement-learning algorithm (DDPG), not a conventional control, and the evaluation covers only three summer months in simulation [25]. Both factors limit the comparability of that record with the rest of the set. Within this sample, Random Forest is the only specific model evaluated in more than one independent real-world study, and both studies report values in a similar range; because the two applications differ in formulation (regression-based predictive control versus classification of thermal sensation from an infrared sensor), outcome (total HVAC demand versus cooling demand), building, and climate, this is an observed pattern rather than a generalizable conclusion. The three generic DRL studies available lie at the low end (3.95–9.7%), although this small number of cases does not allow a performance limit of the DRL family to be established; in addition, the 2025–2026 works show that the DQN and PPO variants report 27–36%, in all cases against non-rule-based baselines or with aggregated attribution of parallel measures, which is consistent with the dispersion being associated with the experimental configuration rather than with the algorithmic family.

Random Forest therefore stands out not because it reports the highest values but because it is, in this sample, the only specific model with two independent real-world evaluations against rule-based baselines. This makes it a candidate for operational transfer that warrants further validation rather than a model of demonstrated superiority; moreover, the sensitivity analysis shows that this distinction is not preserved when the expanded search is considered (Section 3.5). The models with single cases identify configurations with notable results whose generalization requires replication under comparable conditions. Only Zheng et al. (2024) publishes open-source code on the Building Optimization Testing (BOPTEST) framework (1 of 29 studies), which makes it impossible to externally audit the observed dispersion [10]. Nevertheless, even among models with replication, the reported values do not converge uniformly; the same model applied in different studies can produce widely divergent results, and part of that variation may lie in the conditions of the physical environment in which it operates rather than in the model.

### 3.2. Level of Automation and Complexity of Solar-Radiation Management Systems

Twenty-eight studies that evaluated solar-radiation management technologies and dynamic shading systems were considered. Of these, 24 correspond to simulations (86%) and 4 to implementations under real-world conditions (14%), Lee et al. (2023), Lee et al. (2024), Klanatsky et al. (2025), and Humaidan et al. (2025) [29,60,69,70].

To clarify the control approaches identified in the reviewed studies, Table 2 summarizes the main control models, their acronyms, and a brief description of their operational characteristics.

Figure 7 presents this classification, allowing a comparison of how different control strategies are associated with the energy-reduction ranges documented in the literature.

Figure 7 shows that threshold-based controls (TCs), automatic rule-based controls (RBACs), and non-adaptive algorithms (ACAs) concentrate most of their records below 15%, with cases of negative net balance. Seasonal control (SC) and simulation-optimized control (SOAC) are distributed mostly between 10% and 42%, a range that includes the real-world implementation of Humaidan et al. (2025) with 28.4% [70]. Controls optimized through genetic algorithms (GA-OPTs) produce values between 35.9% and 54.7% (Dabbagh et al. 2021) [71]. Automatic multivariable control (MVAC) presents the widest dispersion, from 19.5% (Norouziasas et al. 2023) to 66.1% (Alders 2017) [32,72]. Predictive control (DMPC-PC) brings together four studies, three of them recent simulations that document the transfer of predictive schemes to shading (Roberts et al. 2025), 10% seasonal on polymer-dispersed liquid crystal (PDLC) glass (Liu et al. 2026), 4.1–22.9% on dual-band electrochromic glass across five climates (Wang et al. 2025), 41.3% on photovoltaic shading [73,74,75]. The only documented real-world case of the AI–shading intersection is Klanatsky et al. (2025), which reports a 41% total reduction and 75% in cooling in an annual implementation under real-world conditions [60].

Within each category, the dispersion coincides less with the type of formal scheme than with the nature of the variables that feed it and with the system’s capacity to anticipate changes in load. The Lee et al. [29,30,69] series on the same experimental dwelling in Sejong illustrates this pattern, Lee et al. (2023) reports a 2.1% cooling savings with SPD windows under a fixed tint; and Lee et al. (2024) reports 43% with the same technology, the same dwelling, under illuminance-based control with dimming regulation [29,69]. More than 40 percentage points separate the two configurations with the building and device held constant; however, because the two studies correspond to different measurement campaigns, the difference cannot be attributed exclusively to the control algorithm. Budaiwi et al. (2023) provides the only within-study comparison that isolates the effect of the control variable; on the same EC window, indoor-illuminance control reaches 23% total (cooling 9%, lighting 25%), whereas glare-based control reaches 17% total (cooling 14%, lighting 12%); the 6-percentage-point difference occurs with the device and conditions held constant [37].

In this sample, the type of formal control is an insufficient category for anticipating the reported values; controls of the same category report widely different values, which coincide with differences in the signals they incorporate and the adaptive capacity they enable. What Figure 7 does not differentiate is the physical device on which each control operates. The same threshold strategy reports different results when applied to an exterior curtain or to an optically active window, two technologies whose mechanism of interaction with solar radiation differs structurally.

Table 3 provides a summary of the solar-control technologies identified in the reviewed studies, including their acronyms and operational characteristics.

Nevertheless, the differences observed in energy performance are also associated with the specific characteristics of each shading technology and with its degree of automation. In order to explore this aspect in greater depth, the energy behavior of the main technological solutions for solar-radiation management identified in the literature is analyzed, considering the energy-demand variations reported in the studies that evaluate them. Figure 8 presents this comparison, grouping the results by the type of shading technology implemented.

Figure 8 shows that automatic outdoor curtains (AOCSs) present the highest values, Alders (2017) with 66.1%, Klanatsky et al. (2025) with 41%, Humaidan et al. (2025) with 28.4% total (35.6% in cooling) under real-world conditions, and Firląg et al. (2015) between 6.3% and 25.5% [32,60,70,76]. Louver systems with solar-gain control (LS/GL-SHGC) are concentrated between 35.9% and 54.7% (Dabbagh et al. 2021) [71]. Dynamic photovoltaic overhangs (DPOs) and gasochromic windows (GCWs) produce values between 10% and 35%. Photovoltaic shading devices (PVSDs) reach 41.3% under predictive control (Wang et al. 2025), and PDLC windows (PDLC-Ws) record 10% (Roberts et al. 2025) [73,75]. Automatic indoor curtains (AICSs) report between 6.3% and 34.5%. Electrochromic windows (EC-Ws) present the widest dispersion, from −4% to 29%, with multiple negative heating records. SPD-Ws cover a range from 2.1% to 56% in cooling within the same device, under different control configurations.

In this sample, the physical position of the element in the envelope is associated with the reported values more consistently than the control category. AOCS and LS/GL-SHGC systems intercept radiation before the glazing; the studies that evaluate them tend to concentrate in the upper values of the set. AICS act after radiation passes through the glass and present more bounded reductions. EC-W and SPD-W modify the transmittance of the existing glazing; their wide dispersion is consistent with the need to coordinate the optical state of the device with the building’s load dynamics to produce net savings. The Lee series suggests that the device alone does not determine the outcome; the same SPD in the same building reports between 2.1% and 56% in cooling under different control configurations and measurement campaigns.

In the reviewed studies, the exterior position is associated with high values more frequently than the interior position; although this pattern is not homogeneous, AOCS with threshold control (Firląg 2015) reaches up to 25.5%, whereas AOCS with predictive control (Klanatsky 2025) reaches 75% in cooling [60,76]. The two cases share the device but differ in control logic, input signals, climate, and study design (simulation versus real-world), so the gap cannot be attributed to a single factor. This suggests that the same exterior technology can produce widely different results, and that the magnitude of that difference could vary depending on the operating conditions under which it is evaluated.

### 3.3. Climatic Distribution of AI and Solar-Radiation Management Applications

The climatic context conditions the dominant thermal load and the environmental variability that HVAC systems face. Figure 9 presents the variation in energy demand by climate and functional category, according to the corrected Köppen classification.

Figure 9 shows that studies developed in the temperate climates Cfa, Cfb, and Csa concentrate most of the records and the widest range of values in Cfa runs from 3.95% to 57.4%, in Cfb from 8% to 43.5%, and in Csa from 18% to 43%. Outside the temperate climates, the records become scarcer. BWh brings together three studies with dispersed results: 23% in Park et al. (2024), a 5-day real field test; 6.2% in Mousavi et al. (2023), an annual simulation; and 31% in Maroofiazar & Reveshti (2026), an annual simulation of a hybrid envelope controlled by DQN in the same city as Mousavi (Yazd) [12,14,65]. BSk groups three records dispersed between 7.6% and 51.3%. Dwa brings together three records with a range of 55 percentage points, 7.2% in Sun et al. (2022), 12.27% in Zhu et al. (2025), and 62.4% in Nengroo et al. (2025) [9,25,77]. The cold climates Dfa and Dfb are concentrated between 5.8% and 21.8%.

When the functional category is examined within each climatic group, no climate shows a concentration of a single category. In the temperate climates (Cfa, Cfb, and Csa), the 22 study–climate records are distributed across all functional categories, and the 14 records in the remaining climates also include the prediction (4), optimization (2), prediction + control (4), and control + optimization (4) schemes. The dispersion observed outside the temperate climates therefore cannot be attributed to a particular functional category; with only one to three records per climate in that group, it is not possible to separate the effect of the climate from that of the study design, a distinction that requires more annual studies with comparable baselines in those climates.

The studies with an evaluation horizon of one year or more reveal more stable patterns than the full set. In Cfb, three independent real-world implementations report values within a band of 35–43.5%, Manjarres et al. (2017) with 43.5%, Bünning et al. (2022) with 35%, and Klanatsky et al. (2025) with 41%; it is the only climate with that number of annual real-world studies [21,24,60]. At the climatic extremes, the annual values available fall below 22%, Af records 3.83% (Yau 2018), BWh 6.2% (Mousavi, Yazd), and the two annual studies in Dfb stand at 10.9% and 20.5% [14,19]. The apparent exception is Maroofiazar and Reveshti (2026), with 31% annually in BWh and BSk; their decomposition analysis, however, attributes to the DQN only 30% of the savings of the technological package (approximately 9 percentage points of the base consumption), so that the isolated contribution of the control remains within the pattern [65]. Chaouch et al. (2021) applies the same AFL model to three cities in a single study and reports decreasing values, 32.8% in Csa (Bordeaux), 22.2% in Csa (Istanbul), and 10.9% in Dfb (Klagenfurt); it is the only study that controls for model variation and isolates the effect of climate [16]. The combined pattern is consistent with the hypothesis that the seasonal variability characteristic of temperate climates widens the margin of action for adaptive control, whereas the sustained thermal loads of extreme climates (constant heat or prolonged cold) reduce it; the small number of annual studies outside the temperate climates does not allow this to be established.

In the available evidence, Cfb is the climate with the largest number of independent annual real-world studies (three), which makes it a convenient, although not representative, reference for comparing models. The study by Chaouch et al. (2021) is the only design that isolates the effect of climate on the performance of a specific model, and its results suggest a gradient linking lower seasonal variability with lower savings [16]. Both findings are transferable as experimental-design criteria; future studies in unrepresented climates (tropical, arid, and Southern Hemisphere Mediterranean) should prioritize annual evaluations with comparable baselines to determine whether the observed gradient holds beyond the European and Asian temperate range where the evidence is currently concentrated.

The climatic distribution described shows that widely different values coexist within the same Köppen group. Figure 10 incorporates the specific model as a second dimension on that climatic basis, making it possible to examine whether the dispersion observed within each climate persists when the algorithm is controlled or whether, by contrast, certain models produce stable results regardless of the context in which they are applied.

Figure 10 shows that the climates Cfb and Cfa concentrate the greatest diversity of models. In Cfb, DRL (8% and 9.7%), PINN (9%), AFL (21.3%), ANN + MPC (25.5%), ARMAX (35%), DMPC (41%), and RFR (43.5%) all appear in that climate, with an internal range of 35 points between the lowest and highest values. In Cfa, seven models coexist (DNN, LSTM, RFC, DQN, PPO, ANN, and DRL) with a range from 3.95% to 57.4%. Apart from the generic ANN label, which groups different architectures and studies across five climates, Adaptive Fuzzy Logic and DQN are the specific models with the greatest climatic coverage, AFL appears in Af (3.83%), Cfb (21.3%), Csa (22.2% and 32.8%), and Dfb (10.9%), whereas DQN appears in BSk (31%), BWh (31%), Cfa (22% and 27.3%), and Csa (18%). ANN records six climate–study combinations, 6.2% in BWh, 51.3% in BSk, 23% in BWh, 19.7% in Csa, 25.5% in Cfb, and 52.1% in Cfa. DRL appears twice in Cfb 8% in Lissa et al. (2021) and 9.7% in Kathirgamanathan et al. (2021) [7,64].

Three patterns emerge when crossing the model and climate. First, the two DRL studies in Cfb report close values (8% and 9.7%) despite being applied in a residential building (Lissa) and a large office (Kathirgamanathan) [7,64]. In these two studies, the difference in building type does not alter the result; however, the small number of cases does not allow a performance limit of DRL to be established. Second, AFL and DQN are the only specific models with coverage of four different climates, AFL shows a decreasing gradient from Csa (22.2–32.8%) toward Dfb (10.9%) and Af (3.83%), whereas DQN (Maroofiazar & Reveshti 2026) exhibits the inverse gradient, with 31% in the extreme climates (BSk, BWh) and 18–22% in the temperate ones (Cfa, Csa) [65]. The difference is plausibly related to the controlled domain, since the DQN manages not only the HVAC but an adaptive envelope with radiative cooling whose benefit increases with climatic severity. Third, the ANN in BWh produces results that differ by a factor of 3.7 between Park (23%, real, 5 days) and Mousavi (6.2%, simulation, 1 year) [12,14]. The same model label in the same climate generates incompatible values when the type of study changes, which suggests that, for this climate, the study design weighs more than the algorithm. Random Forest, by contrast, reports close values in two adjacent climates (RFR 43.5% in Cfb, RFC 42% in Cfa), both real-world studies with rule-based baselines, although two cases are insufficient to establish stability across climatic contexts.

The model–climate cross shows that Cfb is the only climate where enough models coexist to compare architectures within the same climatic context; within it, the results range from 8% to 43.5%, a spread that the climate alone cannot account for. Evaluations in Cfb therefore offer a practical setting for comparing models with the climate held constant, although this does not make Cfb representative of other climates. AFL and DQN are the only specific models that allow climatic-sensitivity curves to be traced from existing studies, and their opposite gradients suggest that the direction of that sensitivity may depend on the controlled technological domain (pure HVAC versus adaptive envelope). The specific contribution of this analysis is to identify the model–climate combinations that currently have more than one observation—generic DRL in Cfb (low values), Random Forest in Cfa/Cfb (high values), and AFL and DQN in four climates each (gradients of opposite direction)—which are candidates for replication rather than a basis for generalizable statements.

The same climatic-analysis approach was transferred to solar-radiation management technologies. The 28 studies evaluated their technologies in one or more climatic contexts. Each technology–climate combination was treated as an independent record, forming 78 cases. Figure 11 presents the variation in energy demand by climatic group and type of control.

Figure 11 shows that the widest range of the set is recorded in Cfb. MVAC control with ventilation integration reaches 66.1% (Alders 2017), DMPC-PC reaches 41% (Klanatsky 2025), and threshold controls on EC-W produce values between −9% in heating and 62% in cooling, with a total balance of 16% (Cannavale et al. 2018) [32,39,60]. In BWh, the records range from 25.5% (Firląg 2015) to 54% total (Mesloub et al. 2022), the latter reporting −19% in heating offset by 58% in cooling, and they include the first real-world implementation of the axis in this climate (Humaidan et al. 2025, 28.4%) [70,76,78]. In Dwa, the Lee series produces values from 2.1% to 56% in cooling depending on the algorithm, together with −8% in heating during the winter season (Lee et al. 2025) [30]. In the cold climates Dfb and Dfc, the records are concentrated below 10%, with negative net balances in Piccolo et al. (2018, −4% in Dfb) and Carlucci et al. (2025, −0.7% in Dfc) [31,40].

This climatic analysis of solar-radiation management systems directly addresses research question iii. Considering all 28 studies (Appendix B), the available evidence is concentrated in BWh and Dfb (10 studies each), followed by Dwa and Cfb (7 each), whereas tropical climates (Aw, 2 studies; none in Af or Am), semi-arid climates (BSk and BSh, 1 study each), the subarctic Dfc (1), and the subtropical highland Cwb (2) are barely represented. This limits the possibility of establishing whether the seasonal-penalty pattern described below holds outside the climates currently represented.

The pattern observed across climates is that, in the climatic groups with several records, adaptive controls tend to report the highest values, whereas static controls concentrate the low values and the negative records within that same group. In Cfb, the distance between the highest value (66.1%, MVAC) and the lowest (8%, TC on EC-W from Dussault 2017) exceeds 58 percentage points within a single climate, a difference that, within a single climate, is more plausibly related to the control logic than to the context [79]. The second pattern is the seasonal heating penalty in optically active technologies applied in climates with defined winters, Cannavale et al. (2018) in Csa records −29% in heating with an annual balance of 25%; Oh et al. (2018) in Dwa with seasonal control records −42% in heating with a balance of 24%; Mesloub et al. (2022) in BWh records −19% in heating; and Piccolo et al. (2018) in Dfb reaches −4% total balance [31,39,78,80]. In all these cases, the positive or near-zero annual average conceals a seasonal asymmetry that conditions the operational experience of the building. Carlucci et al. (2025) evaluates EC windows under threshold control, with the thermo-optical properties of the device optimized by a genetic algorithm, in six European locations under five Shared Socioeconomic Pathway (SSP) scenarios (1–2.6 to 5–8.5) [40]. The savings of the optimal device remain at 12–13% in warm climates across the scenarios, whereas a poorly selected device can increase consumption by up to +39% under the most severe scenarios; in Dfc, the balance is negative in all evaluated scenarios.

In the available studies, climate appears to condition the margin of opportunity available for control, but not whether that margin is reached or whether the balance is positive or negative, which appears to depend on the coherence between the control logic and the seasonal regime of the climate. The reported annual averages are insufficient to characterize the performance of technologies that produce savings in summer and penalties in winter; the distinction by the thermal component is the minimum information needed to compare studies across climates. This same variability within each climate suggests that the type of technology modulates the response to the context differently.

The variation of performance between climates makes it possible to identify general trends, but it does not by itself explain the differences observed between the different solar-radiation management technologies. In order to explore how climatic conditions interact with the type of technology used, both factors were integrated into a joint analysis. Figure 12 presents a heat map that synthesizes the median energy-demand variation values reported in the literature for each technology–climate combination, where multiple studies are available. This representation makes it possible to identify patterns of correspondence between the climatic context and the operational response of the different shading solutions, without attempting to establish direct comparisons or causal inferences between technologies.

Figure 12 shows that AOCSs reach the highest values in Cfb (Alders, 66.1%, and Klanatsky, 41%) and present records between 6.3% and 25.5% in the Cfa, BWh, and Dfb climates (Firląg 2015) [32,60,76]. LS/GL-SHGC systems with GA-OPTs are concentrated between 35.9% and 54.7% in Dfb, Csb, BWh, and Dfa (Dabbagh et al. 2021) [71]. GL-SHGCs with SOACs produce between 15% and 42% in Dfb, Csb, and BWh (Krarti 2023) [4]. DPOs report between 16% and 32% in four climates (Krarti 2024) [81]. GCWs produce between 10% and 35% in Cfa and Dwa (Feng 2016) [82]. AICSs present cooling reductions between 19.3% and 34.5% in BWh, Cwb, and Cwa (Kabanshi 2023, not plotted because no total value is reported) and 19.5% in Cfb (Norouziasas 2023) [72,83]. EC-Ws are the technology with the greatest climatic coverage, Dussault (2017) evaluates the same device in 10 climates, with values ranging from 29% in Aw to 5% in Dfb [79]. Cannavale et al. (2023) covers six European cities with the same device, with values between 5% and 13%; and Liu et al. (2026) evaluates a dual-band electrochromic glass under predictive control in five Chinese climates, with values between 4.1% (Dfa) and 22.9% (Cwa) [38,74]. The cold climates Dfb and Dfc group the lowest values of all the technologies, with Piccolo et al. (2018) (−4%, Dfb) and Carlucci et al. (2025) (−0.7%, Dfc) as the only negative annual balances of the set [31,40].

Exterior technologies (AOCSs) and those with direct SHGC control appear to show more stable behavior across climates, plausibly because their mechanism of action intercepts radiation before the glazing, reducing the thermal gain regardless of the seasonal regime. Optically active technologies (EC-W and SPD-W) modify the transmittance of the existing glass and are more sensitive to the seasonal balance; in the reviewed studies, they report their largest savings in climates where the cooling demand dominates the year and heating penalties in climates with pronounced winters, which the heat map reflects in the divergence between their values in BWh and Csa relative to Dfb and Dfc. The study by Dussault (2017) makes it possible to directly compare the behavior of the same EC-W across a diversity of 10 climates, and Liu et al. (2026) replicates the exercise with a dual-band electrochromic glass in five climates [74,79]. In both cases, the values decrease gradually from climates with a high cooling demand toward cold climates, a pattern consistent with the gradient identified in Axis 1 for AFL.

Exterior technologies and those with a controlled SHGC may present greater potential for transfer between climatic contexts because the physical mechanism is less dependent on the seasonal calibration of the control. Optically active technologies require the control to distinguish between periods of beneficial solar gain and periods of overheating; without that distinction, the balance can become negative in cold climates, as observed in the reviewed Dfb and Dfc cases. What the map does not differentiate is which signals the control uses in each technology–climate combination to make that decision, nor how far in advance it incorporates them.

### 3.4. Input Variables in the Models and Their Relationship with Energy Demand

The performance of AI models depends on the set of variables they receive during training and operation. Figure 13 crosses the frequency of occurrence of each variable with the reported energy-variation values.

Figure 13 shows that outdoor temperature and indoor temperature remain the most widely used variables, with 25 and 24 studies (32 and 26 cases), with medians of 21.9% and 21.8% (IQR 10.1–31.5% and 9.2–34.5%, respectively) and ranges spanning from 4% to 62.4%. Solar radiation and occupancy register 13 and 11 mentions, followed by outdoor relative humidity with 9, with similar medians (21.8%, 22.2%, and 19.7%, respectively). Among the five functional families, the anticipatory forecast variables coincide with some of the highest values of the set, although each appears in only three studies (weather conditions: 23%, 41%, and 57.4%; forecast renewable production: 8%, 30.7%, and 62.4%), whereas the hourly electricity price constitutes the exception within that family, with the highest frequency (7 studies) but a median of 12.3% (IQR 9.4–26.0%). The maximum value identified (62.4%) corresponds to a model that combines illuminance, occupancy, hourly electricity price, and renewable production (Nengroo 2025), and the behavioral family is represented by a single study using skin temperature, Levinson et al. (2024, 42%) [20,25]. This relationship between the frequency of use of a variable and the reported savings is associative, not causal: the design of this review does not allow the effect of an individual variable on the energy outcome to be isolated, because each study combines several variables with its own model, climate, and baseline.

The thermal-base variables (indoor and outdoor temperature, and relative humidity) describe the gradient that the HVAC system compensates for and appear in practically all models, which is consistent with their ranges reproducing the full dispersion without discriminating between configurations. Operational variables (occupancy, time, and date) introduce information about internal loads and usage patterns; the analysis shows that the presence of occupancy does not by itself mark a jump. Studies with occupancy report a median of 22.2% (11 studies, 13 cases) versus 22.0% in those that do not include it (18 studies, 23 cases); the highest value with occupancy corresponds to a single case specifically oriented toward it (Liu et al. 2025, 52.1%) [67]. The 2025–2026 studies additionally incorporate measured energy consumption as a feedback signal, grouped with the thermal-base variables in Figure 13 (Liao, Ma & Liu, Liu et al.) [63,67,68]. Anticipatory forecast variables appear in few cases; in the cases available, they coincide with some of the highest savings reported under real-world conditions, 57.4% in Kannan et al. (2020) with weather conditions, 23% in Park et al. (2024) with forecasting, and 41% in Klanatsky et al. (2025) with a 24-h weather forecast and solar-production prediction [11,12,60]. By contrast, the hourly price accompanies demand–response schemes whose reported savings are moderate (7.6% in Jin 2017, 12.3% in Zhu 2025) [23,77].

The emerging functional structure is that thermal variables define the minimum baseline of the control, operational variables expand the decision space without guaranteeing jumps in savings, and the anticipatory forecast and behavioral variables coincide with some of the high values reported in real-world studies, although the number of cases is too small to establish a causal relationship. The distinction within the anticipatory family is the contribution of this analysis; the frequency of a variable does not anticipate its associated performance, since the electricity price is the most widely adopted anticipatory signal yet the one with the lowest median, whereas the physical-forecast signals (weather, renewable production) remain in three studies or fewer despite coinciding with some of the largest reported savings. That low adoption coincides with the prevalence of reactive schemes, and the systematic integration of the five families within a single model remains unexplored, particularly under prolonged experimental conditions.

This same analysis of the input variables was replicated for the solar-radiation management technologies. As in Axis 1, the relationship between the frequency of use of a variable and the reported savings is associative and not causal, since the design of this review does not allow the effect of an individual variable to be isolated from the remaining experimental conditions (model, climate, and device). The variation in performance between technologies and climates is not fully explained by the type of device or by the control scheme, and may also depend on the signals that feed the system’s decision-making at each instant. Figure 14 crosses the frequency of occurrence of each input variable with the energy-variation values reported in the studies.

Indoor illuminance is the most widely used variable, present in 13 of the 28 studies (39 cases), with a median total reduction of 11.4% (IQR 5.5–20.2%). Incident solar radiation appears in 8 studies (7 reporting total demand; 14 cases), with a median of 16.6% (IQR 12.7–24.9%). Indoor temperature registers 7 occurrences and outdoor temperature 5, with medians of 13.0% (13 cases) and 25.5% (11 cases), respectively. Season of the year and total building energy demand appear in 3 studies with similar values. Glare-related variables register 5 occurrences and occupant presence 3; recent studies additionally incorporate solar-geometry variables (Wang et al. 2025) associated with 41.3% under predictive control [75]. At the lower end of the frequency, the weather forecast, solar-production forecast, and indoor ventilation level appear in a single study each, coinciding with some of the highest values (Klanatsky et al. (2025)), which incorporates weather forecasting, solar-production forecasting, and occupant presence, reports 41% [60]. Alders (2017), which combines occupant presence with indoor ventilation level in a control scheme integrated with natural ventilation, reports 66.1% total [32].

The variables are organized into five families that follow the functional logic of Axis 1 (Figure 14). The solar variables (incident and vertical solar radiation, solar angle) and the luminous variables (indoor and outdoor illuminance, glare) dominate in frequency; they describe the instantaneous state of the incoming radiation but neither the thermal dynamics of the building nor the future evolution of the load. Solar variables are grouped separately in this axis because, in shading control, solar radiation is the signal on which the device acts directly, whereas, in Axis 1, it enters as a thermal-load input. The thermal variables (indoor and outdoor temperature, setpoint and window-surface temperatures, building energy demand and consumption, and indoor ventilation level) introduce information about the thermal regime of the building. The operational variables (occupant presence, occupancy schedule, and season of the year) introduce information about use patterns and allow daily or seasonal adjustments, as occupancy, time, and date do in Axis 1. The anticipatory variables (weather forecast and solar-production forecast) appear in a single study (Klanatsky et al. 2025 [60], 41%); in that case, they coincide with a high reported value, although the sample is insufficient to establish a causal relationship. Budaiwi et al. (2023) provides the only within-study comparison that isolates the effect of the control variable with technology and climate held constant, in BWh [37]. The same EC-W controlled by indoor illuminance reaches 23% total (cooling 9%, lighting 25%), whereas glare-based control reaches 17% total (cooling 14%, lighting 12%), a 6-percentage-point difference obtained by changing only the control variable within the same instantaneous luminous family.

The structure of Axis 2 is analogous to that identified in Axis 1: reactive variables predominate in frequency and are associated with moderate savings, whereas anticipatory ones are scarce and coincide with high values. This correspondence between axes, observed in two distinct technological domains, suggests that part of the limitation may lie in the design of the control schemes rather than in the technology, which, in both axes, prioritize easily accessible signals over signals with a greater anticipatory capacity. The specific contribution of this analysis is to identify that the transition from instantaneous luminous variables toward anticipatory variables emerges as a development line with potential in Axis 2, and that the experimental evidence available for that transition is limited to a single real-world study (Klanatsky et al. 2025), whose configuration is not directly replicable without forecasting infrastructure and integrated coordination, although the simulated predictive controls (Roberts, Liu, and Wang) indicate that this transition is already under way at the computational level [73,74,75].

Read from a sensing perspective, the variable families of both axes correspond to different sensor infrastructures. Thermal-base variables rely on conventional temperature and humidity probes already embedded in building management systems; operational variables require occupancy detection, which, in the reviewed studies, ranges from presence sensors to infrared biometric sensing and machine vision (Levinson et al. 2024) and dense Internet of Things (IoT) networks (Ma and Liu 2026) [20,68]; solar and luminous variables in Axis 2 rely on solar-radiation and illuminance sensors and on glare estimation; and anticipatory variables are mostly virtual sensors, that is, weather, solar-production, or price forecasts obtained from external services rather than measured on site. The predominance of reactive variables therefore reflects, at least in part, the sensing infrastructure commonly available, and the limited use of anticipatory signals points to the integration of forecast services and occupancy detection into the control loop as a priority for sensor-to-control research [51,52,53].

### 3.5. Sensitivity Analysis: Expanded Search for Axis 1

The sensitivity search described in Section 2.1 identified 18 additional Axis 1 studies that met the eligibility criteria (Table S1, Figure S1) [84,85,86,87,88,89,90,91,92,93,94,95,96,97,98,99,100,101]. Of the 76 new records screened, 18 were excluded at the title/abstract stage and 23 at full text (15 reported only cost, peak-demand, or flexibility outcomes; 5 did not quantify the energy variation against a baseline; 2 were theoretical or methodological; and 1 addressed a non-eligible building type); 2 reports could not be retrieved, and 15 records were not assessed in full text. The sensitivity analysis is therefore indicative rather than exhaustive.

Several findings of the main analysis are robust to the additional evidence. The median reported reduction remains practically unchanged (22.2%, IQR 9.7–35.9%, *n* = 29, versus 22.2%, IQR 10.1–34.9%, *n* = 47; in this comparison, each study contributes one value, because Table S1 reports one value per study), as does the share of real-world implementations (31% versus 28%), which confirms the predominance of simulation-based evidence. The climatic concentration also persists: 6 of the 18 additional studies were conducted in Cfb, and none in tropical climates.

Two findings of the main analysis are not preserved. First, Random Forest is no longer the only model with more than one independent real-world evaluation: model predictive control (Drgona et al. 2020, 53.5%; Zhu et al. 2025, 12.3%) and data-driven MPC (Klanatsky et al. 2025, 41%; Joe and Karava 2019, 16% in heating) also have at least two [60,77,84,85]. Second, the low values of generic DRL are not confirmed: the seven additional reinforcement-learning studies report reductions between 8.8% and 37% (median 21.4%). Accordingly, the statements on Random Forest and DRL in the main analysis are presented as patterns specific to the original sample, and the Conclusions were adjusted to reflect the expanded evidence.

## 4. Limitations

The findings of this review should be interpreted in light of six limitations, each of which affects the confidence in different conclusions. First, the search was restricted to a single database (Web of Science Core Collection). Studies indexed only in other databases (e.g., Scopus or IEEE Xplore) may have been missed, which mainly affects the completeness of the counts. The sensitivity search, run in the same database, addresses the coverage of the search terms rather than that of the database; it retrieved 18 additional eligible Axis 1 studies, which did not alter the median savings or the share of real-world studies but did modify the statements on replicated models (Section 3.5), and 15 of its records were not assessed in full text. Likewise, some predictive shading-control studies, including several cited in the Introduction, are not part of the corpus because they were not retrieved by the search strings or did not meet the eligibility criteria. The counts reported here should therefore be read as a lower bound.

Second, screening and data extraction were performed primarily by a single reviewer; a second reviewer verified a subsample and the final included set, but no agreement statistic is available. This mainly affects borderline classification decisions (for example, the functional category or the level of automation) and is less likely to affect objective characteristics such as the setting, duration, or climate of each study. Third, no formal scored risk-of-bias or certainty assessment was conducted; the structured appraisal in Appendix C covers the setting, evaluation period, energy outcome, and code availability, but the baseline type and the clarity of attribution were not coded for every study and are discussed only where they condition specific values. The appraisal therefore does not replace a validated instrument, and comparisons between categories should be read as descriptive patterns.

Fourth, the evidence is likely affected by positive-publication bias: studies reporting small or negative savings are less likely to be published, which may inflate the reported ranges, particularly for simulation studies and models supported by a single case. Fifth, most reported savings derive from simulations or short real-world campaigns and implicitly assume reliable sensing, communication, and actuation; the results therefore do not account for the costs, maintenance, or occupant-acceptance barriers that condition technology adoption in practice. Finally, heterogeneity in baselines, durations, outcome definitions, and metrics precluded a meta-analysis and limits the direct comparability of the reported values.

## 5. Conclusions

Regarding the first research question, AI applied to HVAC is dominated by hybrid prediction–control and control–optimization schemes, with reported reductions between 3.8% and 62.4% (median 22.1% across 36 cases from 29 studies). This dispersion coincides with differences in the type of baseline, the duration of the experiment, and the metric reported, so the highest values are not comparable with the rest. In the main sample, Random Forest was the only specific model evaluated in two independent real-world studies with close values (42–43.5%), and the three generic DRL studies lay at the low end (3.95–9.7%); however, neither pattern was preserved in the sensitivity analysis, where MPC and data-driven MPC also showed real-world replication and reinforcement-learning variants reported 8.8–37%. These findings should therefore be read as properties of the available samples rather than as rankings of algorithms.

Regarding the second research question, solar-radiation management systems remain predominantly reactive: 24 of 28 studies are simulations, and threshold, rule-based, and non-adaptive controls concentrate most records below 15% and the negative annual balances. AI-based predictive control of shading is documented in a single real-world study (Klanatsky et al. 2025 [60], 41% total reduction, with partial attribution) and in three recent simulations, so the AI–shading intersection is an emerging line rather than a consolidated field. Comparisons with the device held constant (the Lee et al. [29,30,69] series, with the caveat of separate measurement campaigns, and the within-study comparison of Budaiwi et al. 2023 [37]) suggest that the control logic and its input signals can substantially change the outcome.

Regarding the third research question, the evidence is concentrated in a few climates (Cfa, Cfb, and Csa for HVAC; BWh, Dfb, Dwa, and Cfb for solar management), while tropical, semi-arid, subtropical highland, and subarctic climates are barely represented, and none of the 56 studies was conducted in Latin America. Where evidence exists, optically active technologies show seasonal heating penalties in climates with defined winters that annual aggregates conceal, and single-model multi-climate studies (Chaouch et al. 2021 [16]; Dussault and Gosselin 2017 [79]) suggest lower savings as the seasonal variability decreases. These climatic patterns rest on few studies and require replication with annual evaluations and comparable baselines.

Regarding the fourth research question, both axes share the same structure of input variables: reactive thermal and luminous variables provided by conventional sensors dominate, operational variables such as occupancy do not by themselves change the median savings, and anticipatory variables, mostly virtual sensors based on forecasts, appear in three studies or fewer but coincide with some of the highest reported values. This association is not causal, but it identifies the integration of forecast services and occupancy detection into the control loop as a relevant direction for sensor-to-control research.

Overall, the main cross-cutting finding is the asymmetry of experimental maturity between axes (31% real-world studies in HVAC versus 14% in solar management), together with the limited comparability of the reported results. The first priority for future research is therefore to transfer predictive and data-driven control from HVAC to solar management through real-world validations of at least one year, with rule-based baselines and disaggregated attribution when several measures operate in parallel, particularly in currently unrepresented climates. Secondary priorities are to report results by thermal component (heating, cooling, lighting, and total) so that seasonal penalties become visible, to publish code and simulation models to allow external auditing, and to evaluate the sensing infrastructure required by anticipatory control, including its cost and reliability, as part of the energy assessment. For practice and policy, these priorities translate into requiring a declared baseline and component-level reporting in the performance assessment of automated HVAC and shading controls, and into supporting pilot deployments in the climates and regions currently absent from the evidence, such as Latin America.

## Figures and Tables

**Figure 1:**
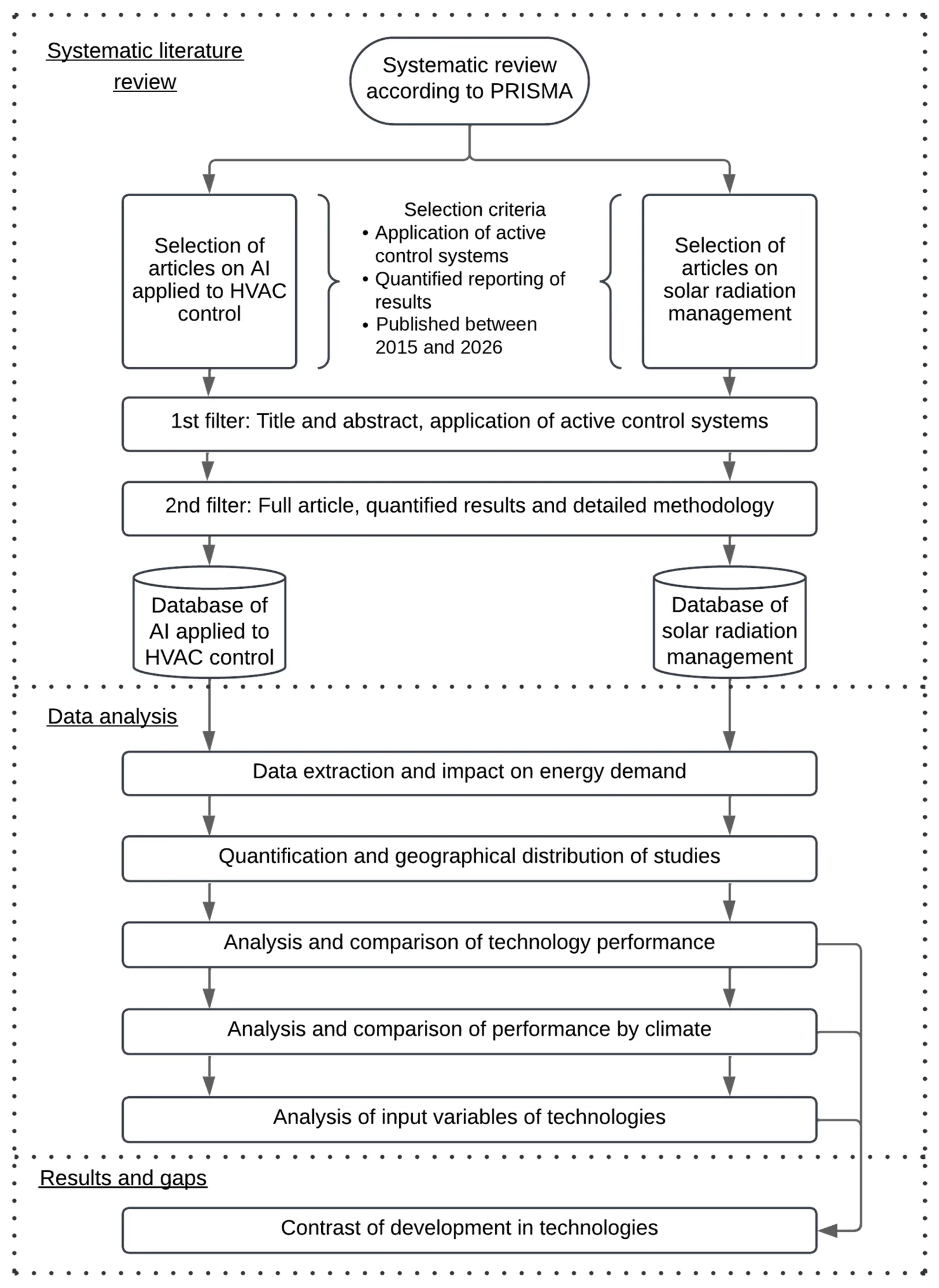
Workflow of the systematic review and of the comparative analysis of the two axes (AI-based HVAC control and solar-radiation management).

**Figure 2:**
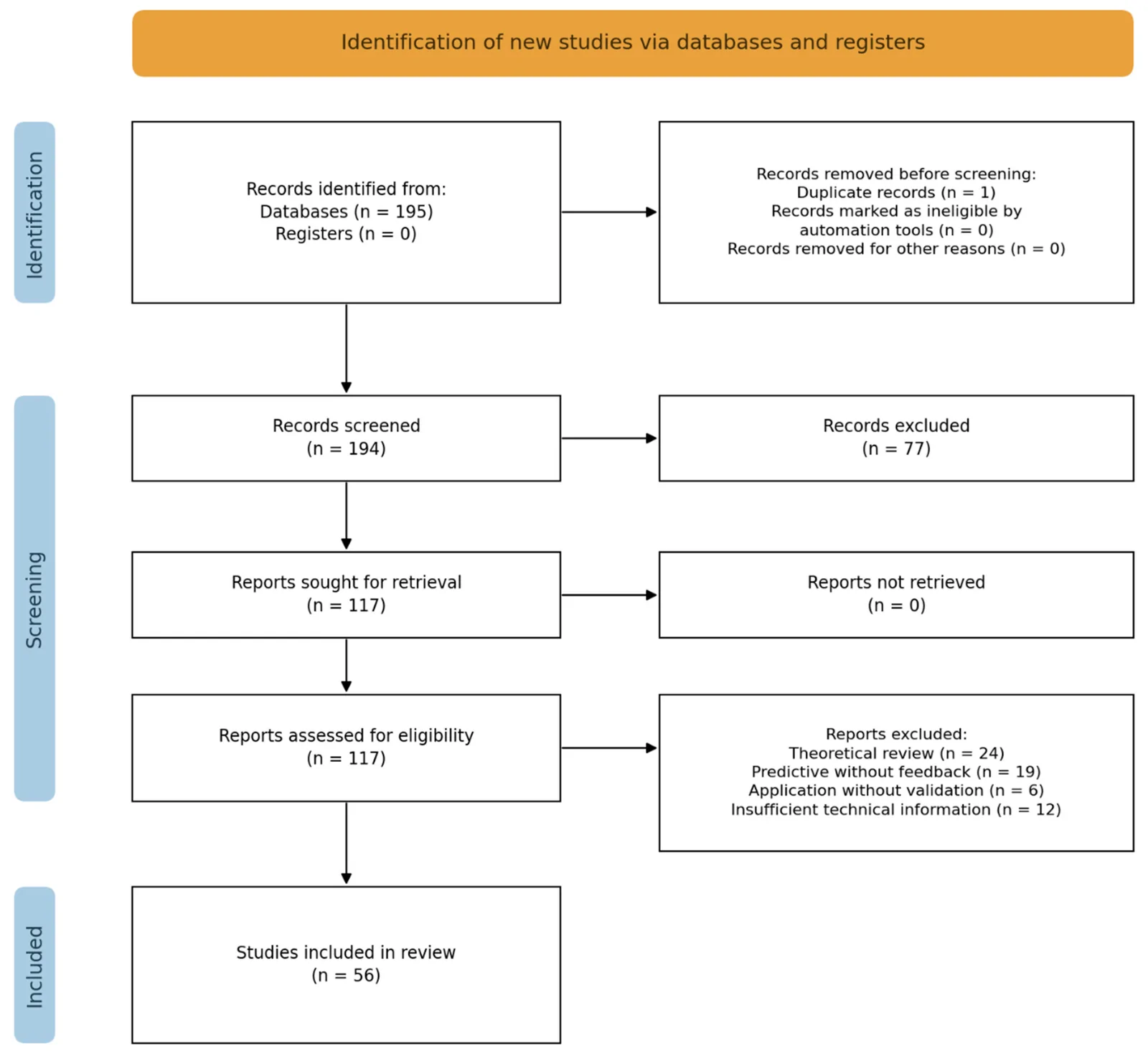
Article selection (PRISMA flow diagram).

**Figure 3:**
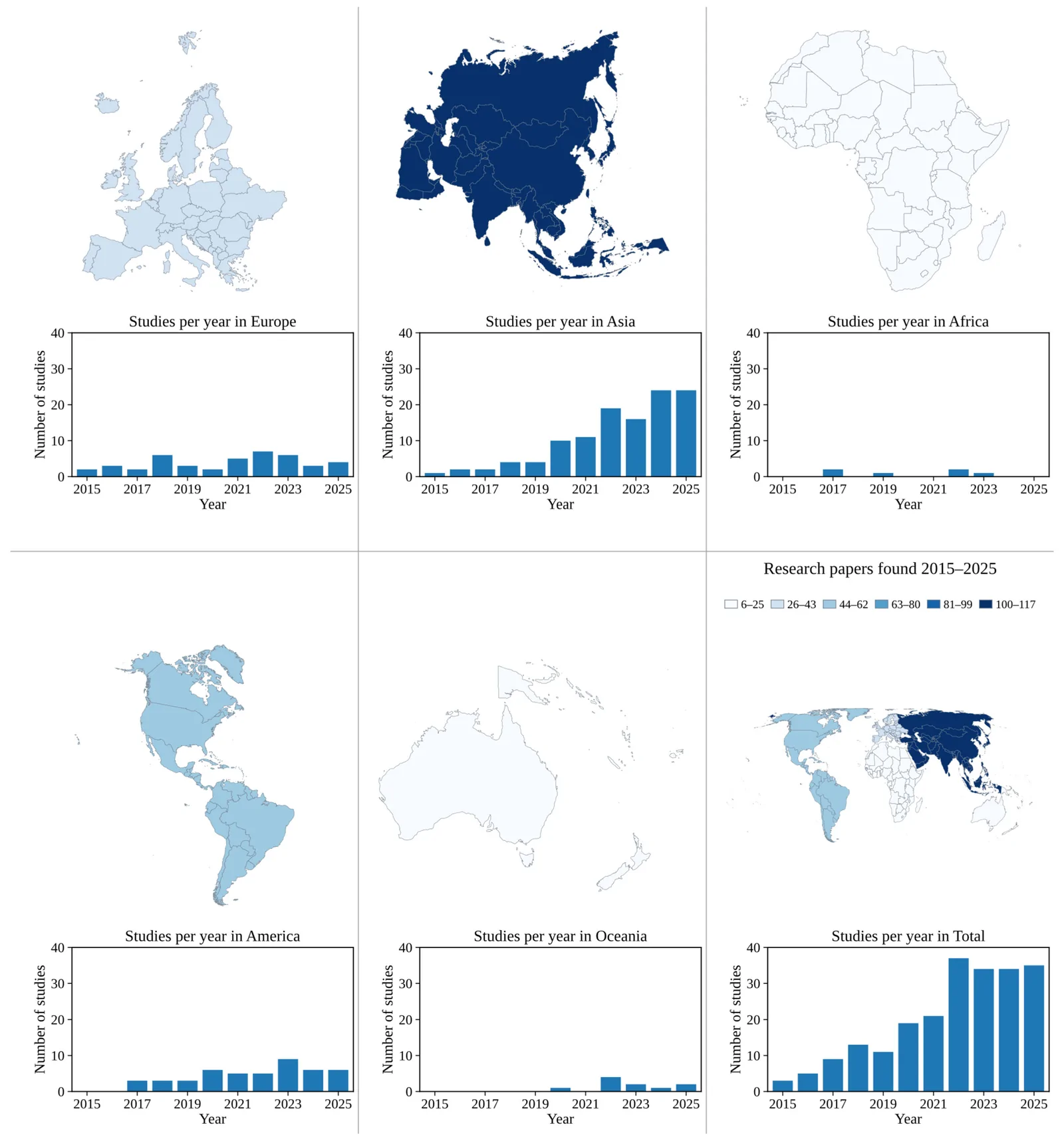
Geographical distribution and annual number of the records retrieved in Web of Science.

**Figure 4:**
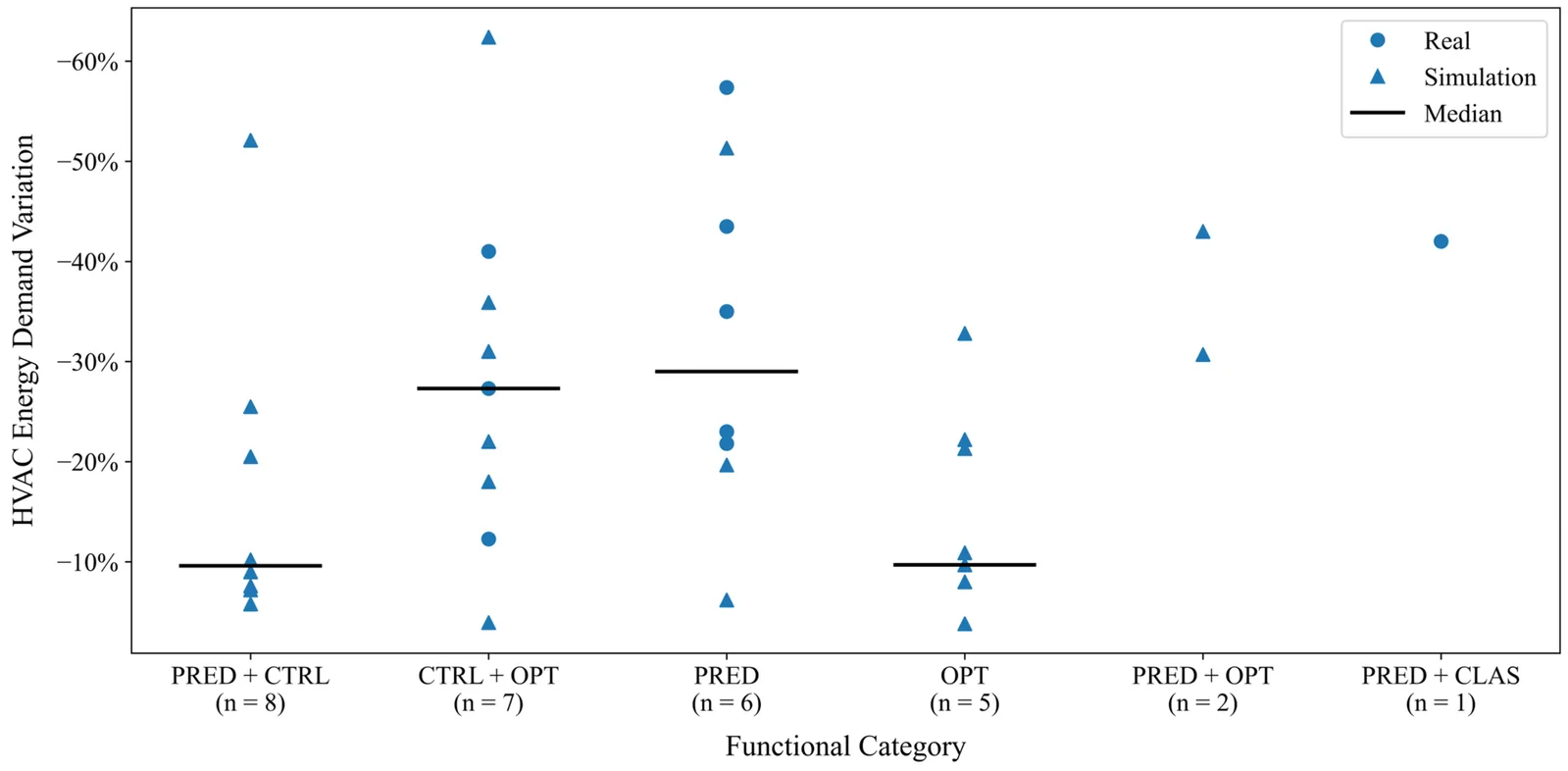
Energy-demand variations reported for AI-based HVAC control, by functional category (Axis 1). Each marker is one case (a study–climate or study–configuration value); circles denote real-world and triangles simulation studies; *n* = number of independent studies; the horizontal line marks the median for groupings with five or more cases; reductions are plotted as negative values.

**Figure 5:**
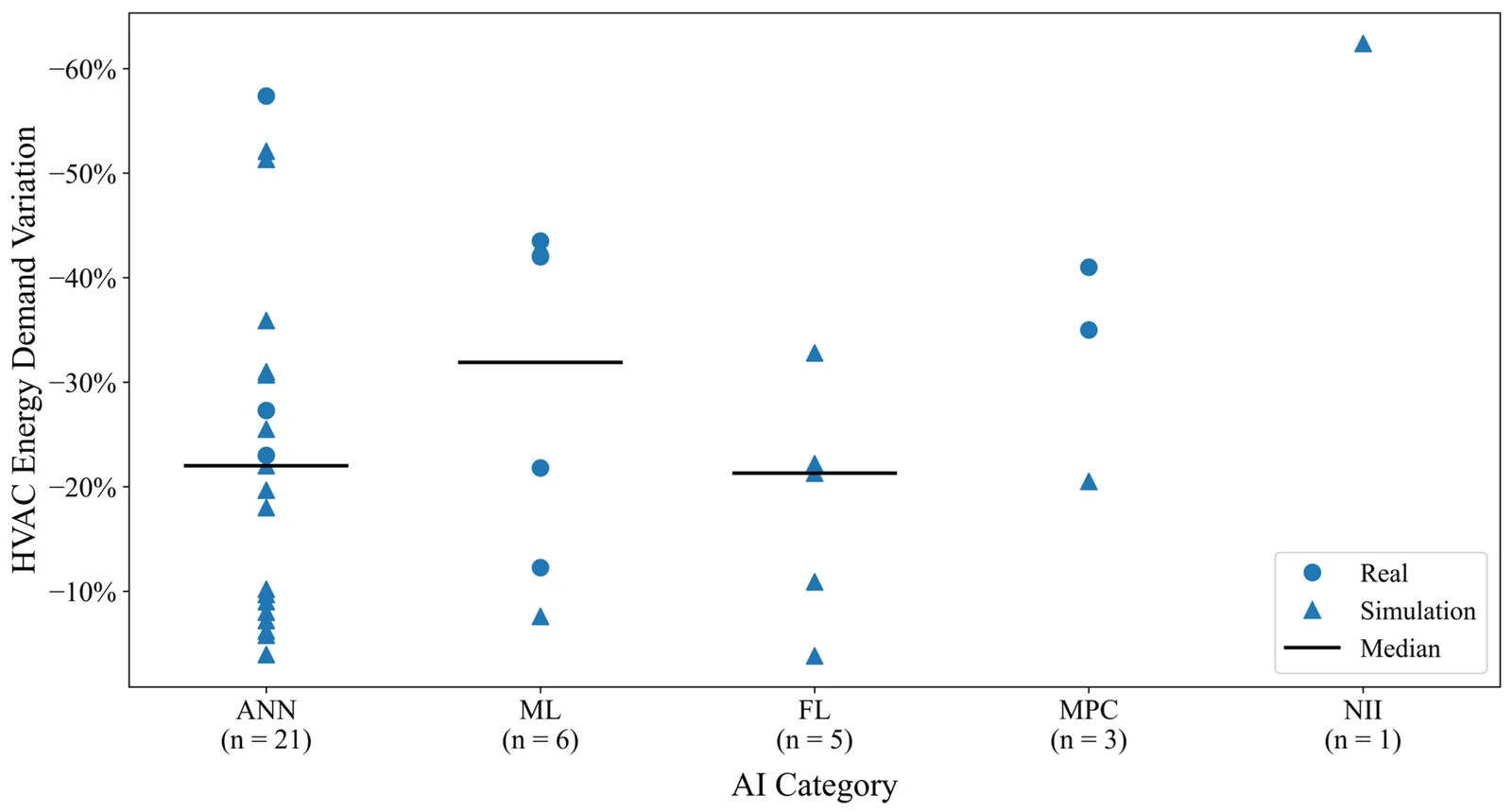
Energy-demand variations reported for AI-based HVAC control, by AI technique (Axis 1). ANNs: artificial neural networks; ML: machine learning; FL: fuzzy logic; MPC: model predictive control; NII: nature-inspired intelligence. Markers, *n*, and medians as in Figure 4.

**Figure 6:**
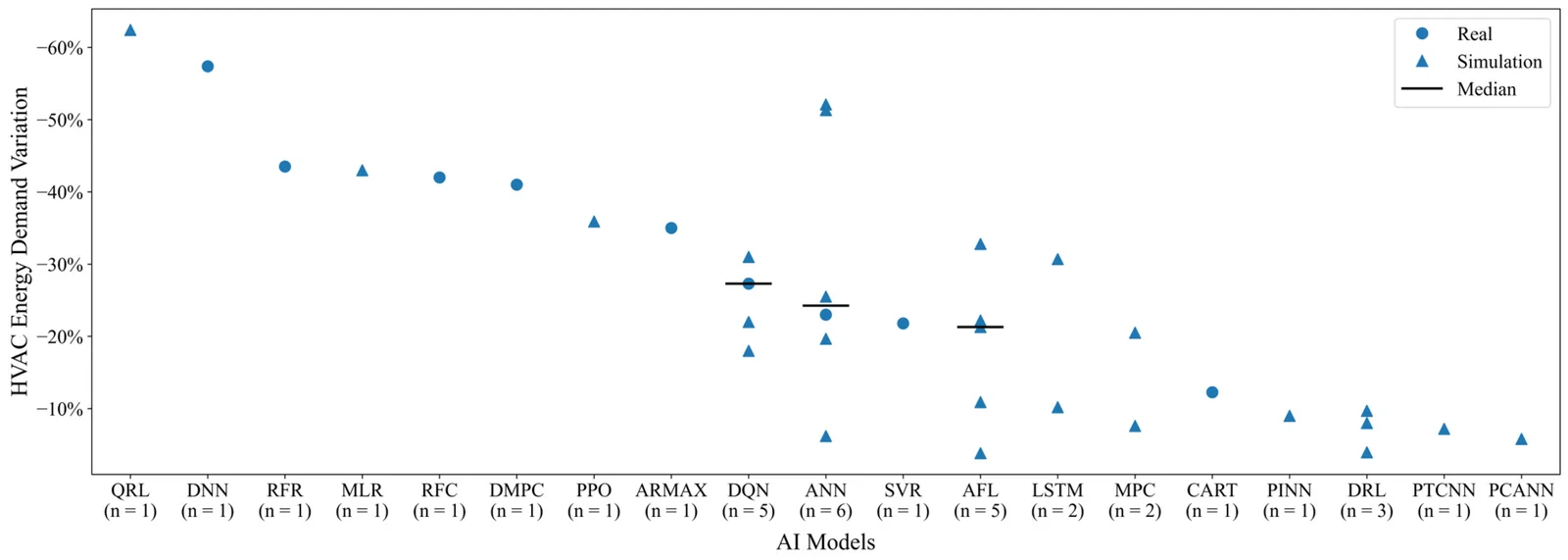
Energy-demand variations reported for AI-based HVAC control, by specific AI model (Axis 1). Model acronyms as in Table 1; markers, *n*, and medians as in Figure 4.

**Figure 7:**
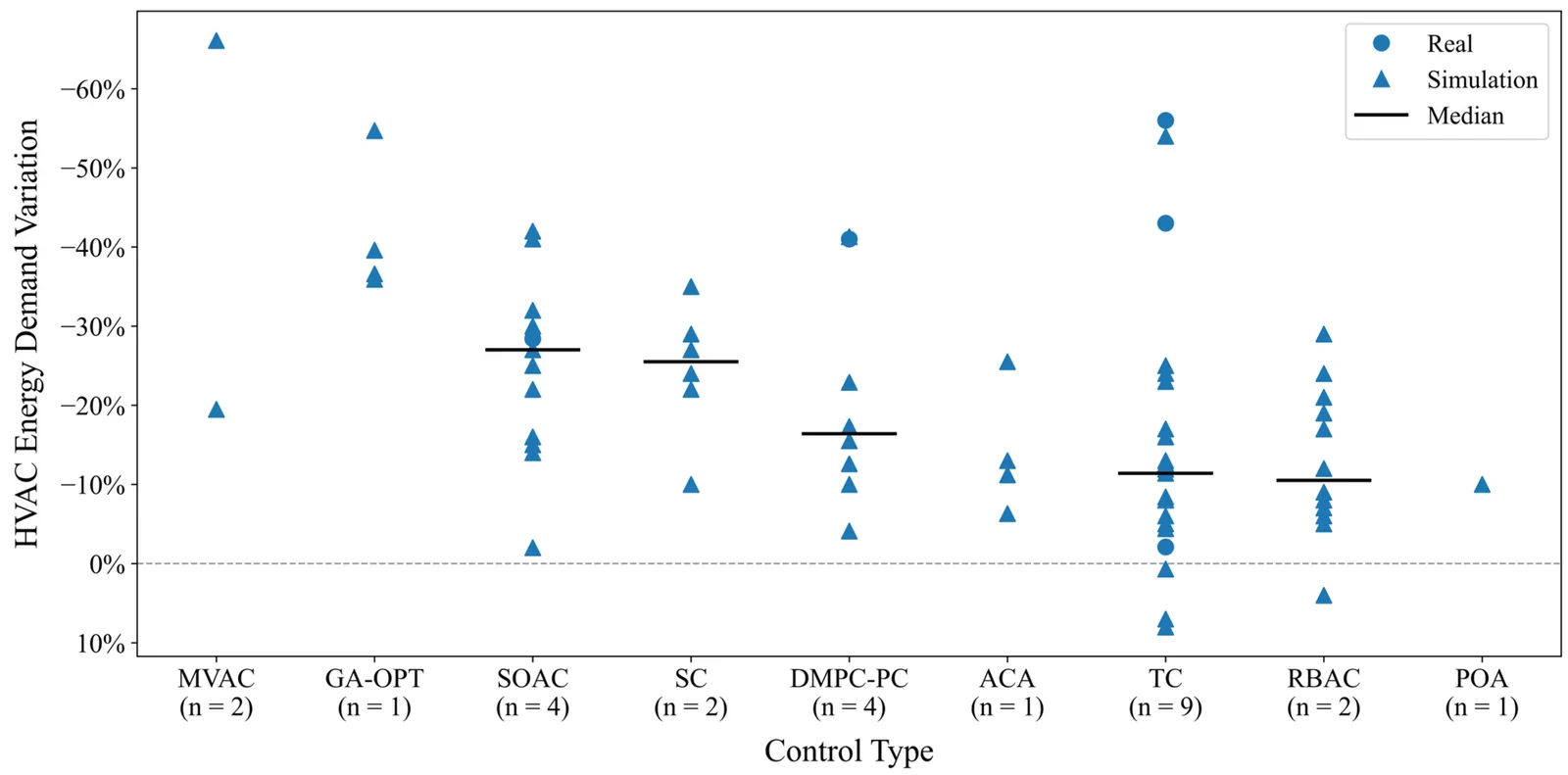
Energy-demand variations reported for solar-radiation management, by type of control (Axis 2). Control acronyms as in Table 2. Each marker is one case of total HVAC demand; circles denote real-world and triangles simulation studies; *n* = number of independent studies; the horizontal line marks the median for groupings with five or more cases; reductions are plotted as negative values. Two studies that report only heating or cooling values are listed in Appendix B but not plotted.

**Figure 8:**
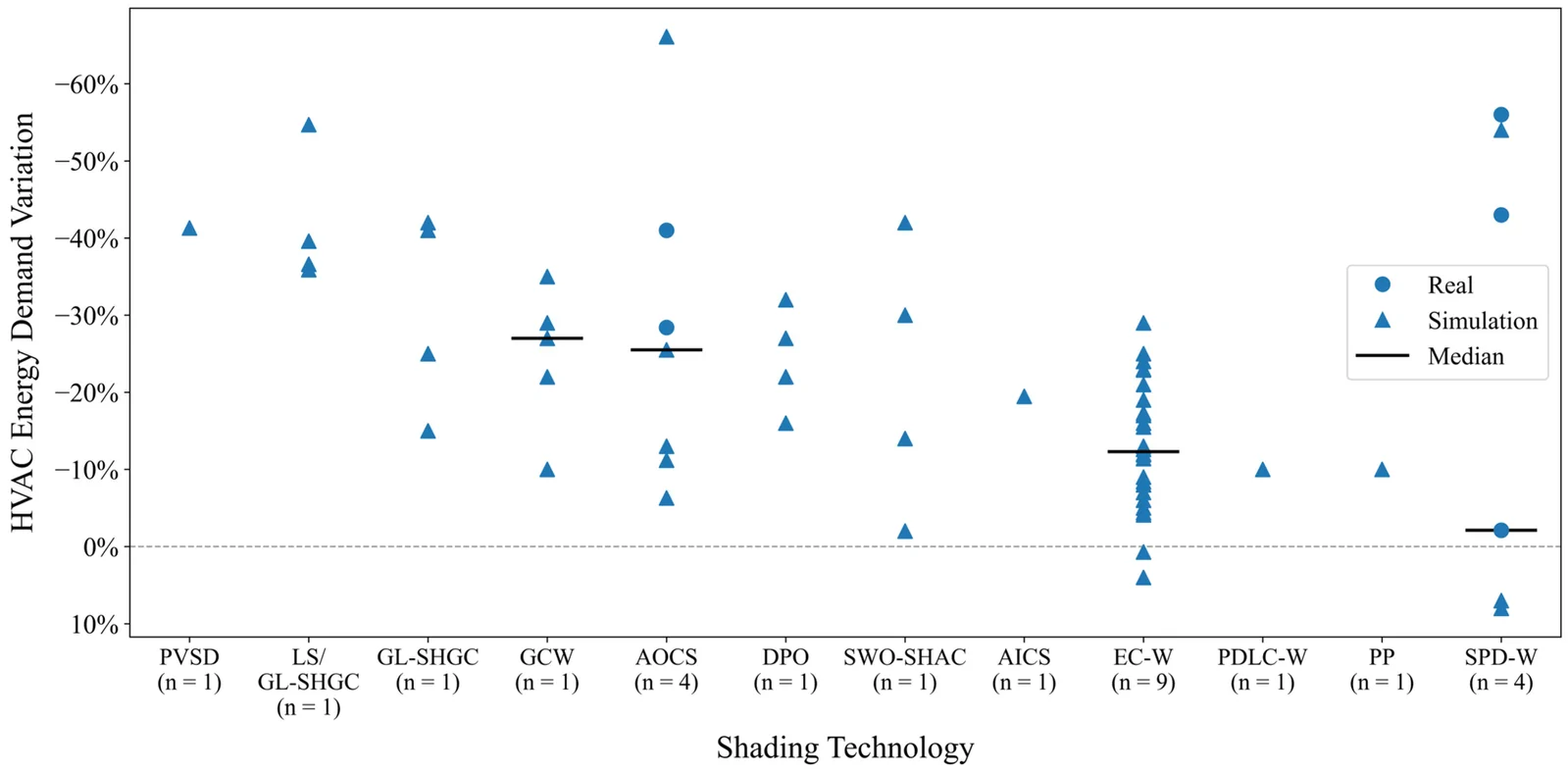
Energy-demand variations reported for solar-radiation management, by shading technology (Axis 2). Technology acronyms as in Table 3; markers, *n*, and medians as in Figure 7.

**Figure 9:**
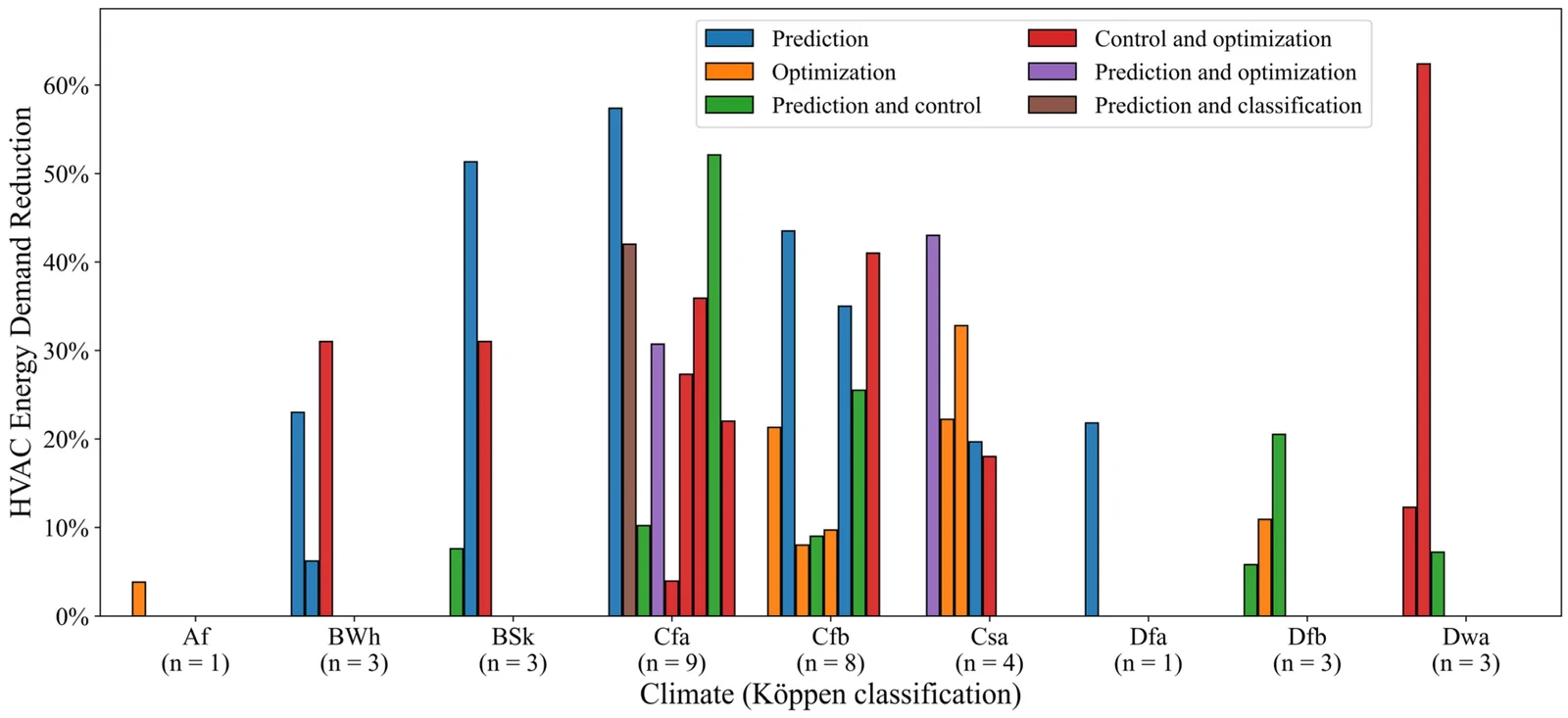
Energy-demand reductions reported for AI-based HVAC control, by Köppen climate and functional category (Axis 1). Each bar is one case; *n* = number of independent studies per climate; reductions are shown as positive values.

**Figure 10:**
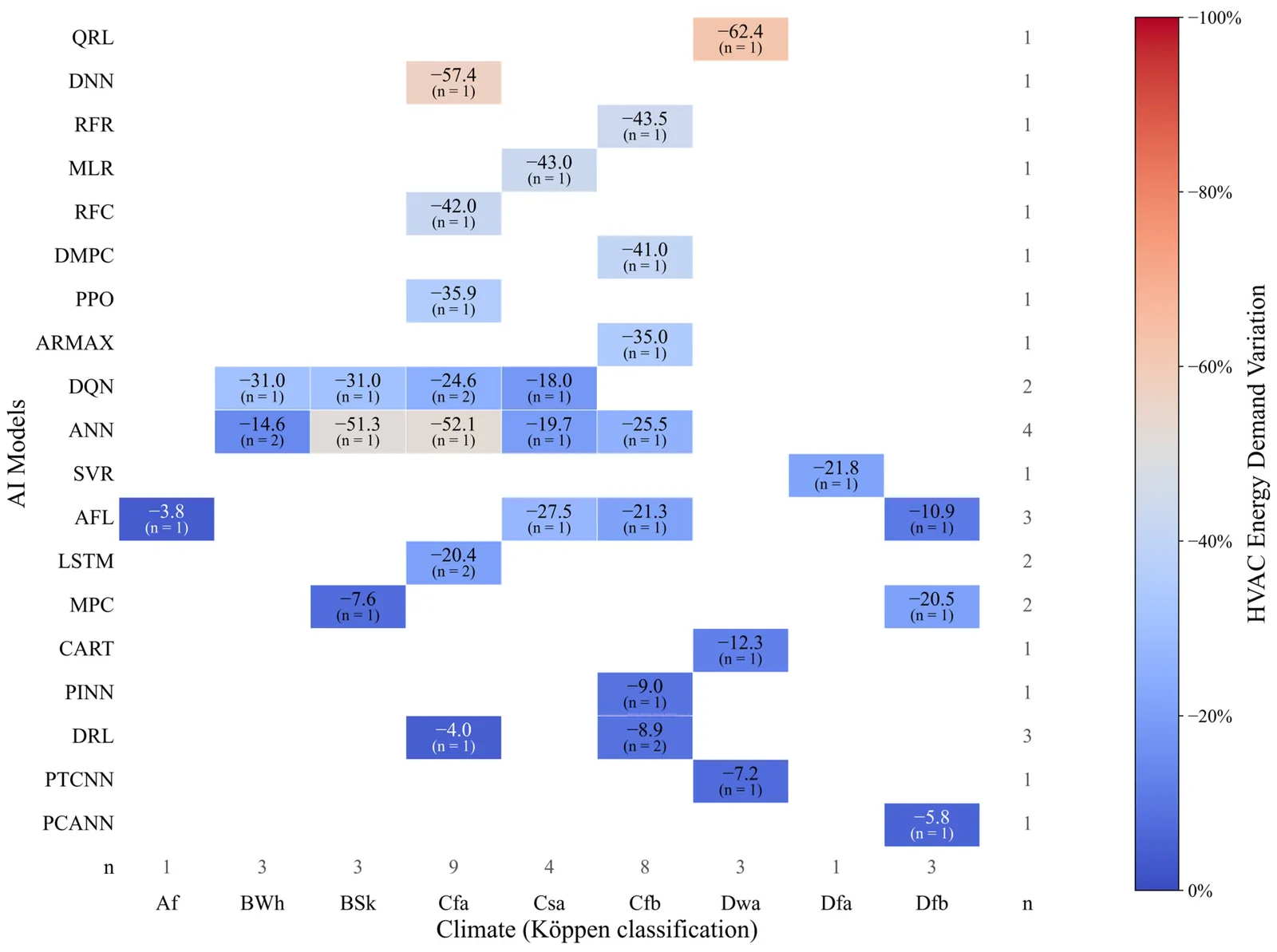
Median energy-demand variation by AI model and Köppen climate (Axis 1). Each cell shows the median of the cases in that model–climate combination; *n* (in cells and margins) = number of independent studies; reductions are shown as negative values.

**Figure 11:**
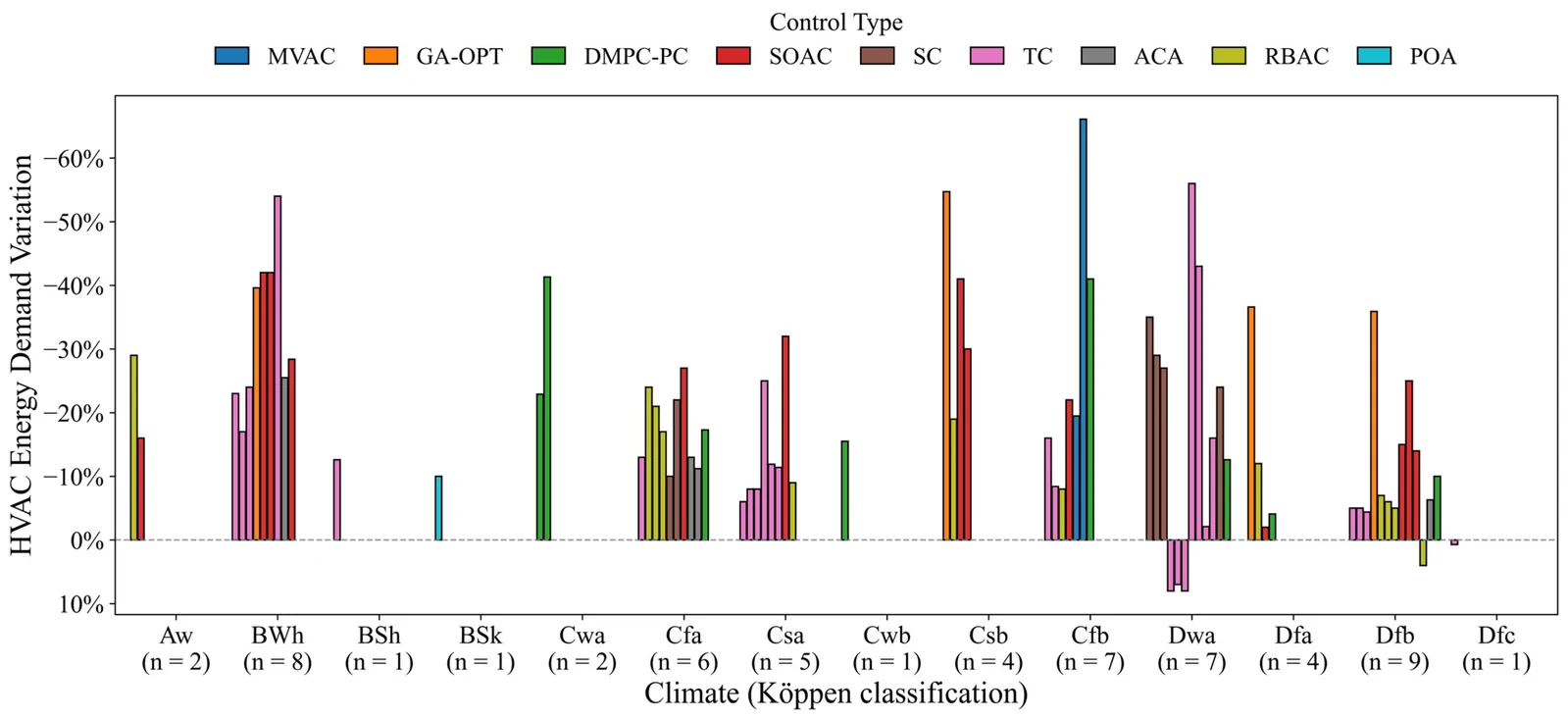
Energy-demand variations reported for solar-radiation management, by Köppen climate and type of control (Axis 2). Each bar is one case of total HVAC demand; *n* = number of independent studies per climate; control acronyms as in Table 2; reductions are plotted as negative values.

**Figure 12:**
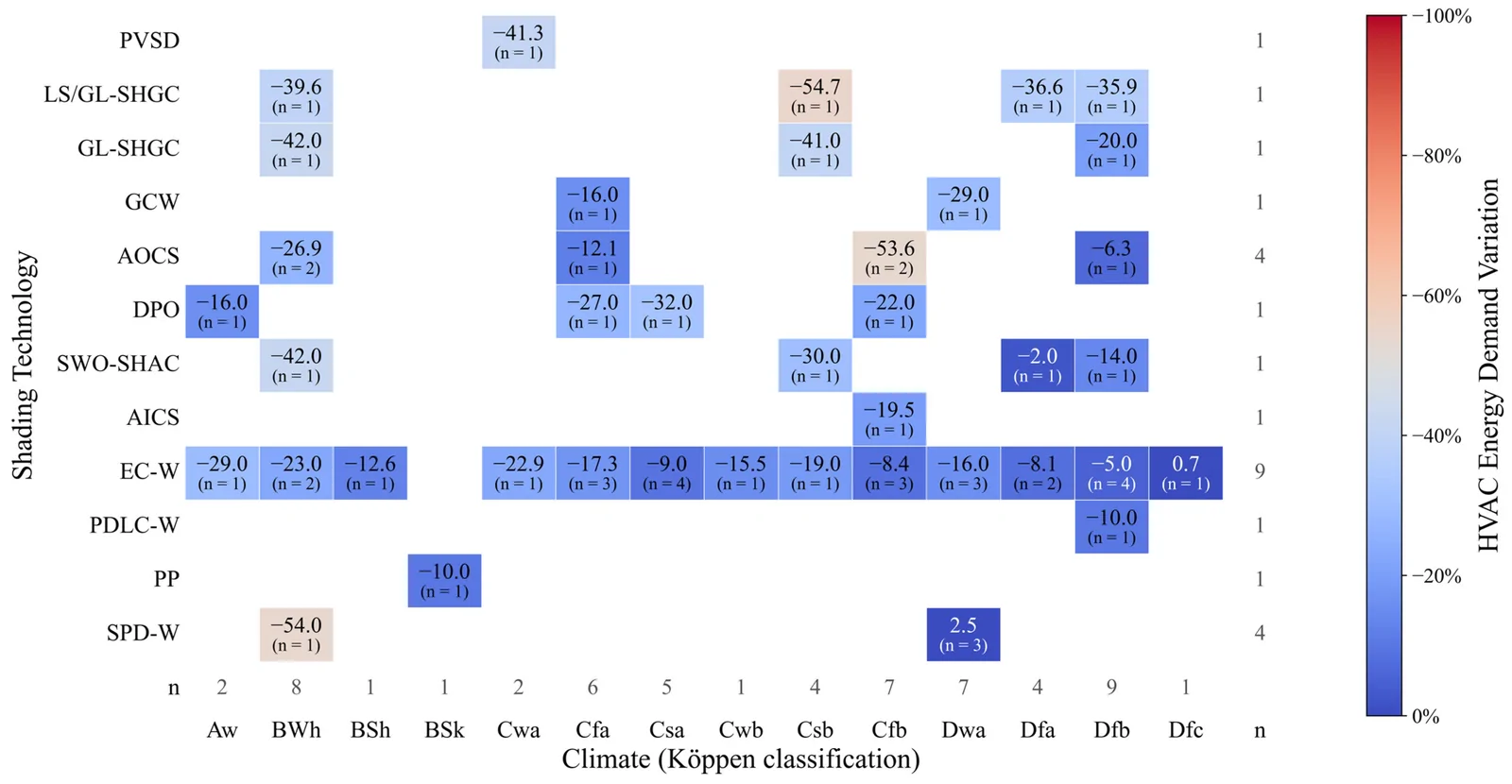
Median energy-demand variation by shading technology and Köppen climate (Axis 2). Each cell shows the median of the cases of total HVAC demand in that technology–climate combination; *n* (in cells and margins) = number of independent studies; technology acronyms as in Table 3; reductions are shown as negative values.

**Figure 13:**
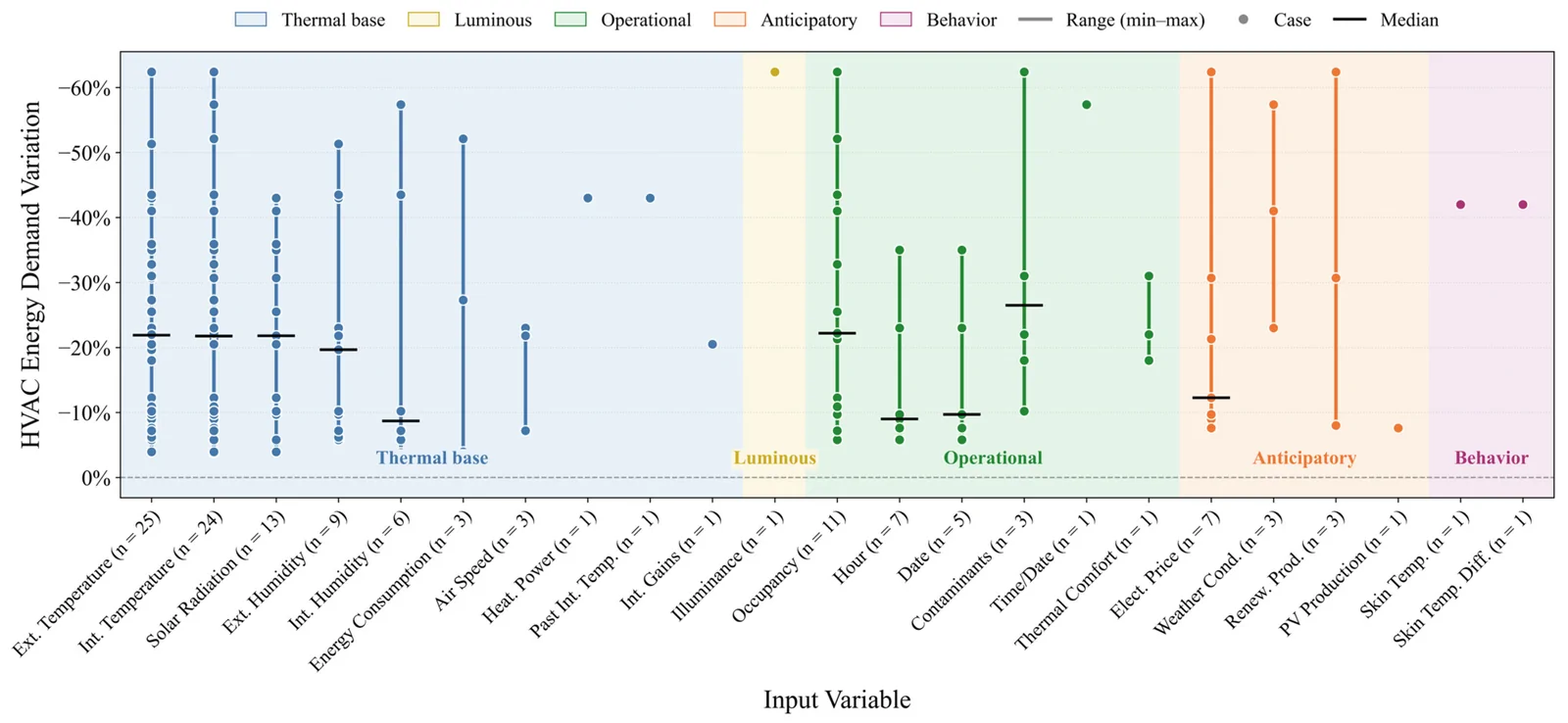
Input variables of AI-based HVAC control models and the associated energy-demand variations, grouped by functional family (Axis 1). Vertical lines show the range, markers the individual cases, and horizontal lines the median for variables with five or more cases; *n* = number of studies using each variable. The association is descriptive and does not imply a causal effect of the variable.

**Figure 14:**
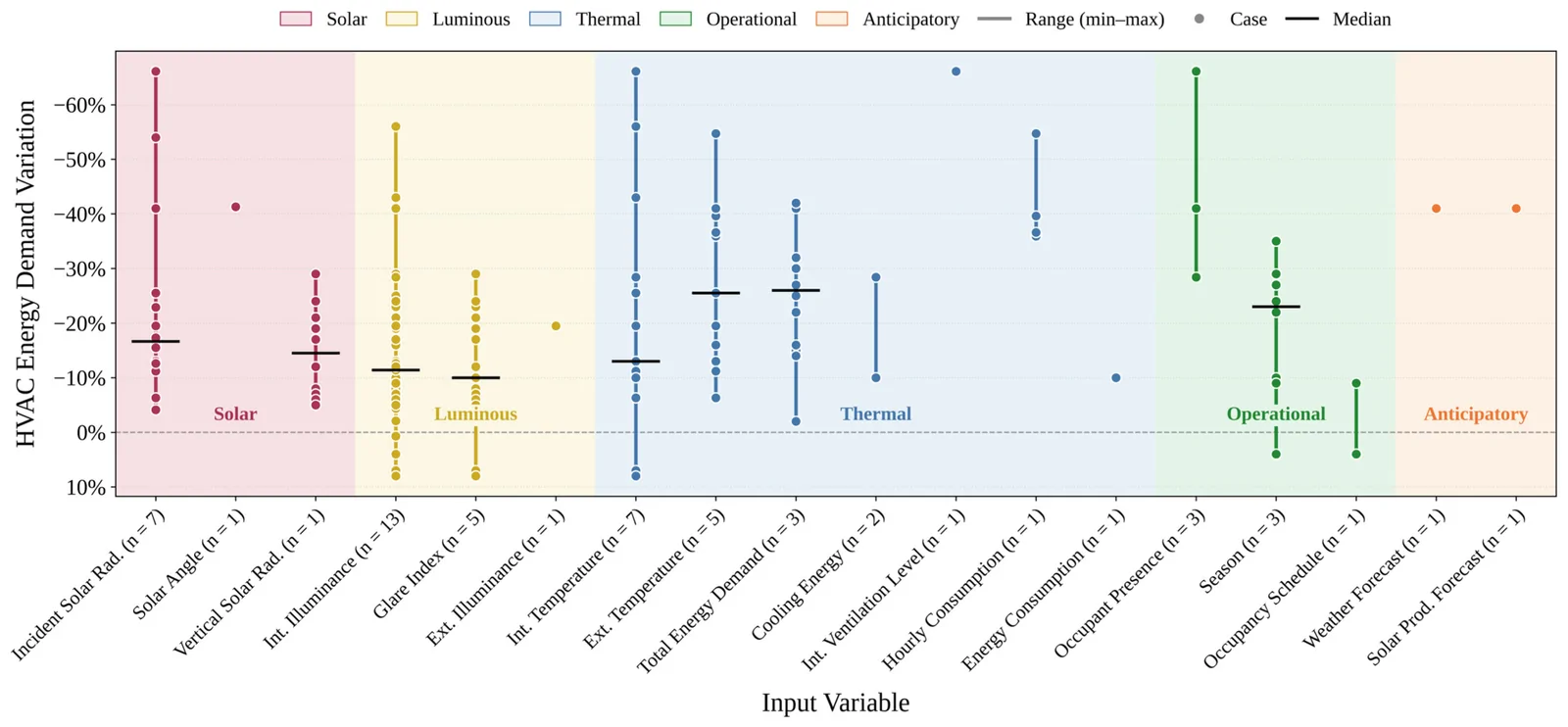
Input variables of solar-radiation management controls and the associated variations in total HVAC demand, grouped by functional family (Axis 2). Symbols and *n* as in Figure 13; the association is descriptive and does not imply a causal effect of the variable.

**Table 1:** Summary of AI model types.

Acronym	Model Name	Brief Description
QRL	Quantum Reinforcement Learning	Reinforcement learning approach that incorporates quantum computing principles to enhance exploration of the decision space.
DNN	Deep Neural Network	Deep architecture with multiple hidden layers used to model complex nonlinear relationships.
RFR	Random Forest Regression	Ensemble of regression trees that improves accuracy through random sampling of features and data.
MLR	Multiple Linear Regression	Statistical linear model that relates multiple predictor variables to a continuous output variable.
RFC	Random Forest Classification	Classification method based on multiple decision trees that increases stability and reduces overfitting.
DMPC	Data-driven Model Predictive Control	MPC variant that uses data-driven models to forecast and optimize system operation.
ARMAX	Autoregressive Moving Average with Exogenous Inputs	Autoregressive model that incorporates external variables to describe dynamic systems.
ANN	Artificial Neural Network	Classical neural network architecture trained to approximate functions through supervised learning.
SVR	Support Vector Regression	Regression method based on optimal margin maximization using kernel functions to capture nonlinear relationships.
LSTM	Long Short-Term Memory	Recurrent neural network architecture designed to capture long-term temporal dependencies.
AFL	Adaptive Fuzzy Logic	Fuzzy logic system that adjusts its control rules according to operational conditions.
MPC	Model Predictive Control	Predictive control strategy that optimizes system operation based on explicit future behavior forecasts.
CART	Classification and Regression Trees	Decision-tree method that partitions the data space using hierarchical rules.
PINN	Physics-Informed Neural Network	Neural network that embeds physical constraints directly into the loss function to improve model consistency.
DRL	Deep Reinforcement Learning	Reinforcement-learning method using deep networks to learn optimal policies.
PTCNN	Parallel Temporal Convolutional Neural Network	Temporal convolutional network with parallel structures, used to model multi-scale temporal sequences.
PCANN	Physically Consistent Artificial Neural Network	ANN variant that incorporates physical consistency constraints into its architecture or training to improve stability and interpretability.
DQN	Deep Q-Network	Deep-reinforcement-learning algorithm that approximates the action-value (Q) function with a deep neural network to learn discrete control policies.
PPO	Proximal Policy Optimization	Policy-gradient reinforcement-learning algorithm that optimizes the control policy using a clipped surrogate objective to ensure stable updates.

**Table 2:** Control models and acronyms.

Acronym	Model Name	Brief Description
MVAC	Multivariable Automatic Control	Control strategy that manages multiple interacting variables within the system simultaneously.
GA-OPT	Genetic Algorithm Optimized Control	Control approach that uses genetic algorithms to optimize operational parameters.
DMPC-PC	DMPC-based Predictive Control	Predictive control method based on data-driven Model Predictive Control.
SOAC	Simulation-Optimized Automatic Control	Control strategy optimized through simulation-based performance evaluation.
SC	Seasonal Control	Control method that adjusts operational parameters based on seasonal conditions.
TC	Threshold Control	Simple rule-based control that activates responses when predefined thresholds are exceeded.
ACA	Automatic Control Algorithm	General automatic control scheme using predefined computational rules for system operation.
RBAC	Rule-Based Automatic Control	Control strategy structured around predefined logical rules for system decision-making.
POA	Parametric Optimization Algorithm	Algorithm that adjusts system parameters through optimization procedures to improve performance.

**Table 3:** Solar control systems and acronyms.

Acronym	System Name	Brief Description
LS/GL-SHGC	Louvers System/Glass (SHGC)	Combination of louvers and glazing designed to regulate solar heat gain.
GL-SHGC	Glass (SHGC)	Glazing system characterized by its solar heat gain coefficient.
AOCS	Automatic Outdoor Curtain System	Exterior curtain system that operates automatically to modulate solar radiation.
AICS	Automatic Indoor Curtain System	Interior curtain system with automatic actuation for solar control.
GCW	Gasochromic Window	Glazing that modulates transmittance through gas-induced chromic reactions.
DPO	Dynamic Photovoltaic Overhangs	Overhangs integrating photovoltaic modules with dynamic positioning.
PVSD	Photovoltaic Shading Device	Adjustable shading elements integrating photovoltaic modules, whose position is set by the control strategy.
SWO-SHAC	Smart Windows and Overhangs (SHAC)	Smart adaptive glazing and overhang systems used within SHAC configurations.
SPD-W	SPD Windows	Electro-optical windows using Suspended Particle Devices for dynamic tinting.
EC-W	Electrochromic Window	Smart glazing capable of modulating transmittance based on electrochromic reactions.
PDLC-W	PDLC Window	Glazing with a polymer-dispersed liquid crystal layer that switches between diffuse and transparent states.
PP	Perforated Panels	Panels with perforated patterns designed to reduce incident solar radiation.

## Data Availability

The data extracted from the reviewed studies are included within the article (Appendix A, Appendix B and Appendix C) and the Supplementary Materials (Table S1). Further inquiries can be directed at the corresponding author.

## Supplementary Materials

The following supporting information can be downloaded at: https://www.mdpi.com/article/10.3390/s26206405/s1, Figure S1: PRISMA flow diagram of the sensitivity search; Table S1: Studies included in the sensitivity analysis; PRISMA 2020 checklist. The full Web of Science search strategies and applied filters are reported in Section 2.1. The studies listed in Table S1 are cited in the reference list [84,85,86,87,88,89,90,91,92,93,94,95,96,97,98,99,100,101].

## Appendix A. Studies on AI Applied to HVAC Management Systems

**Author**	**AI Category**	**AI Type**	**Function Performed**	**Environment**	**Reported Energy Variation [%] (Total HVAC Unless Otherwise Indicated)**
Keshtkar et al. (2017) [18]	Fuzzy Logic	Adaptive fuzzy logic	Optimization	Simulation	Heating 21.3%
Kannan et al. (2020) [11]	Artificial Neural Networks	Deep Neural Network (DNN)	Prediction	Real	Cooling 57.4%
Nengroo et al. (2025) [25]	Nature-Inspired Intelligence, Artificial Neural Networks	Quantum Reinforcement Learning, Artificial Neural Networks	Control and optimization	Simulation	Cooling 62.4%
Yu et al. (2025) [15]	Artificial Neural Networks, Model Predictive Control	Long Short-Term Memory, Physically Consistent Neural Network, Hierarchical Model Predictive Control	Prediction and optimization	Simulation	30.7%
Lissa et al. (2021) [7]	Artificial Neural Networks	Deep Reinforcement Learning (DRL)	Optimization	Simulation	Heating 8%
Park et al. (2024) [12]	Artificial Neural Networks	Artificial Neural Network	Prediction	Real	Cooling 23%
Ławryńczuk et al. (2019) [22]	Model Predictive Control	Model Predictive Control	Prediction and control	Simulation	Heating 20.5%
Sha et al. (2025) [61]	Artificial Neural Networks, Model Predictive Control	Long Short-Term Memory, Model Predictive Control	Prediction and control	Simulation	10.2%
Bünning et al. (2022) [24]	Model Predictive Control	Autoregressive Moving Average with Exogenous Variables	Prediction	Real	35%
Pavirani et al. (2024) [8]	Artificial Neural Networks, Nature-Inspired Intelligence	Physics-Informed Neural Network, Monte Carlo Tree Search (MCTS)	Prediction and control	Simulation	Heating 9%
Jin et al. (2017) [23]	Model Predictive Control, Machine Learning	Model Predictive Control, Multiple Linear Regression, Clustering and patterns	Prediction and control	Simulation	7.6%
Sun et al. (2022) [9]	Artificial Neural Networks, Nature-Inspired Intelligence	Parallel Temporal Convolutional Neural Network, Improved Chimp Optimization Algorithm (ICHOA)	Prediction and control	Simulation	7.2%
Zheng et al. (2024) [10]	Artificial Neural Networks, Model Predictive Control	Artificial Neural Networks, Model Predictive Control	Prediction and control	Simulation	25.5%
Klanatsky et al. (2025) [60]	Model Predictive Control	Data-driven Model Predictive Control	Control and optimization	Real	41%
Mousavi et al. (2023) [14]	Artificial Neural Network	Artificial Neural Network	Prediction	Simulation	6.2–51.3%
Amasyali et al. (2021) [102]	Machine Learning	Support Vector Regression (SVR)	Prediction	Real	Cooling 21.8%
Yau et al. (2018) [19]	Fuzzy Logic	Adaptive fuzzy logic	Optimization	Simulation	Cooling 3.8%
Levinson et al. (2024) [20]	Machine Learning, Artificial Neural Networks	Random Forest Classification, Machine Vision (MediaPipe Holistic)	Prediction and classification	Real	Cooling 42%
Chaouch et al. (2021) [16]	Fuzzy Logic	Adaptive fuzzy logic	Optimization	Simulation	10.9–32.8%
Kathirgamanathan et al. (2021) [64]	Artificial Neural Networks	Deep Reinforcement Learning	Optimization	Simulation	Cooling 9.7%
Xiao et al. (2023) [62]	Artificial Neural Networks, Model Predictive Control	Physically Consistent Deep Learning (PCDL), Model Predictive Control	Prediction and control	Simulation	5.8%
Manjarres et al. (2017) [21]	Machine Learning	Random Forest Regression	Prediction	Real	43.5%
Abdellatif et al. (2022) [13]	Machine Learning, Nature-Inspired Intelligence	Multiple Linear Regression, Genetic Algorithm	Prediction and optimization	Simulation	Heating 43%
Zhu et al. (2025) [77]	Model Predictive Control, Machine Learning	Model Predictive Control, Classification and Regression Trees	Control and optimization	Real	Cooling 12.3%
Liao et al. (2025) [63]	Artificial Neural Networks	Deep Reinforcement Learning (DRL)	Control and optimization	Simulation	4%
Ma & Liu (2026) [68]	Artificial Neural Networks	Deep Q-Network (DQN)	Control and optimization	Real	27.3%
Zhang et al. (2026) [66]	Artificial Neural Networks	Proximal Policy Optimization (PPO)	Control and optimization	Simulation	35.9%
Liu et al. (2025) [67]	Artificial Neural Networks	Convolutional Neural Network (CNN)	Prediction and control	Simulation	52.1%
Maroofiazar & Reveshti (2026) [65]	Artificial Neural Networks	Deep Q-Network (DQN)	Control and optimization	Simulation	18–31%

## Appendix B. Solar Radiation Management Studies

**Author**	**Solar Control Device**	**Level of Automation**	**Environment**	**Reported Energy Variation [%] (Total Unless Otherwise Indicated)**
Lee et al. (2025) [30]	SPD windows	Threshold control	Simulation	Heating −8 to −7%
Lee et al. (2024) [69]	SPD windows	Threshold control	Real	Cooling 43–56%
Lee et al. (2023) [29]	SPD windows	Threshold control	Real	Cooling 2.1%
Krarti (2022) [34]	Smart windows and overhangs (SHAC)	Simulation-optimized automatic control	Simulation	2–42%
Krarti (2023) [4]	Glass (SHGC)	Simulation-optimized automatic control	Simulation	15–42%
Krarti et al. (2024) [81]	Dynamic photovoltaic overhangs	Simulation-optimized automatic control	Simulation	16–32%
Firląg et al. (2015) [76]	Automatic outdoor curtain system	Automatic control algorithm	Simulation	6.3–25.5%
Alders (2017) [32]	Automatic outdoor curtain system	Multivariable automatic control	Simulation	66.1%
Dabbagh et al. (2021) [103]	Glass (SHGC)	Rule-based automatic control	Simulation	Heating 0–47%; cooling 28.6–59.8%
Dabbagh et al. (2021) [71]	Louvers system/Glass (SHGC)	Genetic Algorithm optimized control	Simulation	35.9–54.7%
Kabanshi et al. (2023) [83]	Automatic indoor curtain system	Threshold control	Simulation	Cooling 19.3–34.5%
Klanatsky et al. (2025) [60]	Automatic outdoor curtain system	DMPC-based predictive control	Real	41%
Piccolo et al. (2018) [31]	EC window	Rule-based automatic control	Simulation	−4 to 9%
Oh et al. (2018) [80]	EC window	Seasonal control	Simulation	24%
Oh et al. (2018) [104]	EC window	Threshold control	Simulation	16%
Norouziasas et al. (2023) [72]	Automatic indoor curtain system	Multivariable automatic control	Simulation	19.5%
Mesloub et al. (2022) [78]	SPD windows	Threshold control	Simulation	54%
Feng et al. (2016) [82]	Gasochromic window	Seasonal control	Simulation	10–35%
Dussault et al. (2017) [79]	EC window	Rule-based automatic control	Simulation	5–29%
Carlucci et al. (2025) [40]	EC window	Threshold control	Simulation	−0.7 to 12.6%
Cannavale et al. (2018) [39]	EC window	Threshold control	Simulation	16–25%
Cannavale et al. (2023) [38]	EC window	Threshold control	Simulation	5–13%
Budaiwi et al. (2023) [37]	EC window	Threshold control	Simulation	17–23%
Bazazzadeh et al. (2021) [105]	Perforated panels	Parametric optimization algorithm	Simulation	10%
Humaidan et al. (2025) [70]	Automatic outdoor curtain system	Simulation-optimized automatic control	Real	28.4%
Roberts et al. (2025) [73]	PDLC window	DMPC-based predictive control	Simulation	10%
Liu et al. (2026) [74]	EC window	DMPC-based predictive control	Simulation	4.1–22.9%
Wang et al. (2025) [75]	Photovoltaic shading device (PVSD)	DMPC-based predictive control	Simulation	41.3%

## Appendix C. Structured Quality Appraisal of the Included Studies

The table below summarizes, for each of the 56 included studies, the setting, evaluation period, energy outcome, and code availability used to qualify the comparability of the reported energy variations (Section 2.1); the baseline type and attribution clarity are discussed in the text (Section 3.1, Section 3.2, Section 3.3 and Section 3.4). The appraisal is descriptive: no study was excluded or weighted on its basis.

**Study**	**Axis**	**Setting**	**Evaluation Period**	**Energy Outcome**	**Code**
Keshtkar et al. (2017) [18]	1	Simulation	1 month	H	N
Kannan et al. (2020) [11]	1	Real	2 years	C	N
Nengroo et al. (2025) [25]	1	Simulation	3 summer months	C	N
Yu et al. (2025) [15]	1	Simulation	3 months	T	N
Lissa et al. (2021) [7]	1	Simulation	8 months	H	N
Park et al. (2024) [12]	1	Real	5 days	C	N
Ławryńczuk et al. (2019) [22]	1	Simulation	1 year	H	N
Sha et al. (2025) [61]	1	Simulation	1 week	T	N
Bünning et al. (2022) [24]	1	Real	2 years	T	N
Pavirani et al. (2024) [8]	1	Simulation	11 days	H	P
Jin et al. (2017) [23]	1	Simulation	1 week	T	N
Sun et al. (2022) [9]	1	Simulation	Days (not specified)	T	P
Zheng et al. (2024) [10]	1	Simulation	6 months	T	O
Klanatsky et al. (2025) [60]	1, 2	Real	1 year	T	N
Mousavi et al. (2023) [14]	1	Simulation	1 year	T	N
Amasyali et al. (2021) [102]	1	Real	3 months	C	P
Yau et al. (2018) [19]	1	Simulation	1 year	C	P
Levinson et al. (2024) [20]	1	Real	5 months	C	N
Chaouch et al. (2021) [16]	1	Simulation	1 year	T	N
Kathirgamanathan et al. (2021) [64]	1	Simulation	1 summer week	C	N
Xiao et al. (2023) [62]	1	Simulation	14 days	T	P
Manjarres et al. (2017) [21]	1	Real	1 year	T	N
Abdellatif et al. (2022) [13]	1	Simulation	1 year	H	N
Zhu et al. (2025) [77]	1	Real	3 summer weeks	C	P
Liao et al. (2025) [63]	1	Simulation	1 year	T	N
Ma & Liu (2026) [68]	1	Real	9 months	T	N
Zhang et al. (2026) [66]	1	Simulation	2 h	T	N
Liu et al. (2025) [67]	1	Simulation	1 day	T	N
Maroofiazar & Reveshti (2026) [65]	1	Simulation	1 year	T	N
Lee et al. (2025) [30]	2	Simulation	Winter season	H	—
Lee et al. (2024) [69]	2	Real	1 summer month	C	—
Lee et al. (2023) [29]	2	Real	Summer season	C	—
Krarti (2022) [34]	2	Simulation	1 year	T	—
Krarti (2023) [4]	2	Simulation	1 year	T	—
Krarti et al. (2024) [81]	2	Simulation	1 year	T	—
Firląg et al. (2015) [76]	2	Simulation	1 year	T	—
Alders (2017) [32]	2	Simulation	1 year	T	—
Dabbagh et al. (2021) [103]	2	Simulation	1 year	H + C	—
Dabbagh et al. (2021) [71]	2	Simulation	1 year	T	—
Kabanshi et al. (2023) [83]	2	Simulation	1 year	C	—
Piccolo et al. (2018) [31]	2	Simulation	1 summer month	T	—
Oh et al. (2018) [80]	2	Simulation	1 year	T	—
Oh et al. (2018) [104]	2	Simulation	1 year	T	—
Norouziasas et al. (2023) [72]	2	Simulation	1 year	T	—
Mesloub et al. (2022) [78]	2	Simulation	1 year	T	—
Feng et al. (2016) [82]	2	Simulation	1 year	T	—
Dussault et al. (2017) [79]	2	Simulation	1 year	T	—
Carlucci et al. (2025) [40]	2	Simulation	1 year	T	—
Cannavale et al. (2018) [39]	2	Simulation	1 year	T	—
Cannavale et al. (2023) [38]	2	Simulation	1 year	T	—
Budaiwi et al. (2023) [37]	2	Simulation	1 year	T	—
Bazazzadeh et al. (2021) [105]	2	Simulation	1 year	T	—
Humaidan et al. (2025) [70]	2	Real	1 year	T	—
Roberts et al. (2025) [73]	2	Simulation	Typical weeks (summer/winter)	T	—
Liu et al. (2026) [74]	2	Simulation	1 year	T	—
Wang et al. (2025) [75]	2	Simulation	1 year	T	—
Setting: Real, real-world implementation; Simulation, simulated evaluation. Energy outcome: T, total HVAC demand; H, heating only; C, cooling only; H + C, heating and cooling reported separately without a total. Code: O, open code; P, model or method described in sufficient detail for partial replication; N, not available; —, not extracted (Axis 2).					
