# Supplementary material for: Intelligent Systems for Space Conditioning and Shading in Buildings: A Systematic Review of Artificial Intelligence Techniques and Their Energy Impact
Source: Sensors (Basel). 2026 Oct 10;26(20):6405. doi: 10.3390/s26206405 (PMC13708935; doi:10.3390/s26206405)
Supplement: Supplementary file 1 [file sensors-26-06405-s001.zip › Checklist_PRISMA_2020_sensors-4500105.pdf]

# PRISMA 2020 Checklist

*Manuscript sensors-4500105 — Intelligent systems for space conditioning and shading in buildings: a systematic review*

Note: Items 11 and 18 (risk of bias) and 20a were not addressed through a formal, scored instrument; instead, the study-level quality dimensions (baseline type, evaluation duration, real-world vs. simulation, attribution clarity, metric completeness) were extracted and incorporated into the synthesis, and the absence of a standardized assessment is acknowledged in Section 4. Section numbers should be finalized against the resubmitted manuscript.

| Section and Topic | Item | Checklist item | Location where item is reported |
|---|---|---|---|
| **TITLE** | | | |
| Title | **1** | Identify the report as a systematic review. | Title page |
| **ABSTRACT** | | | |
| Abstract | **2** | See the PRISMA 2020 for Abstracts checklist. | Abstract; registration status reported in Methods (Section 2.1) |
| **INTRODUCTION** | | | |
| Rationale | **3** | Describe the rationale for the review in the context of existing knowledge. | Section 1 |
| Objectives | **4** | Provide an explicit statement of the objective(s) or question(s) the review addresses. | Section 2 (four research questions) |
| **METHODS** | | | |
| Eligibility criteria | **5** | Specify the inclusion and exclusion criteria and how studies were grouped for the syntheses. | Section 2.1 |
| Information sources | **6** | Specify all databases and other sources searched, and the date each was last searched. | Section 2.1 (Web of Science Core Collection; searched 20 January 2026) |
| Search strategy | **7** | Present the full search strategies for all databases, including any filters and limits. | Section 2.1 (full Boolean strategies for both axes) |
| Selection process | **8** | Specify how many reviewers screened each record, whether independently, and automation tools used. | Section 2.1 (single reviewer; second reviewer screened a random sample of 35 records and reassessed the included set) |
| Data collection process | **9** | Specify how many reviewers collected data, whether independently, and automation tools used. | Section 2.1 (single reviewer; no duplicate extraction; no automation tools) |
| Data items | **10a** | List and define all outcomes for which data were sought. | Section 2.1 (percentage change in energy demand) |
| Data items | **10b** | List and define all other variables for which data were sought. | Sections 2.1–2.3 (technique, climate, variables, baseline, duration, metric) |
| Study risk of bias assessment | **11** | Specify the methods used to assess risk of bias in the included studies. | *No standardized RoB instrument applied; structured quality appraisal by domains (baseline type, evaluation duration, real-world vs. simulation, attribution clarity, metric completeness) reported in Section 2.1; absence of a formal scored assessment acknowledged in Section 4.* |
| Effect measures | **12** | Specify the effect measure(s) used in the synthesis or presentation of results. | Section 2.1 |
| Synthesis methods | **13a** | Describe the processes used to decide which studies were eligible for each synthesis. | Section 2.1 |
| Synthesis methods | **13b** | Describe any methods required to prepare the data for presentation or synthesis. | Section 2.1 |
| Synthesis methods | **13c** | Describe any methods used to tabulate or visually display results. | Sections 3.1–3.4 (tables, scatter plots, heat maps) |
| Synthesis methods | **13d** | Describe any methods used to synthesize results and the rationale. | Section 2.1 (descriptive synthesis, informed by SWiM; no meta-analysis) |

| | | | |
|---|---|---|---|
| Synthesis methods | **13e** | Describe any methods used to explore possible causes of heterogeneity. | Sections 3.1–3.4 |
| Synthesis methods | **13f** | Describe any sensitivity analyses. | Section 2.1 (none performed) |
| Reporting bias assessment | **14** | Describe methods to assess risk of bias due to missing results. | Section 2.1 (not formally assessed; acknowledged as a limitation) |
| Certainty assessment | **15** | Describe methods to assess certainty in the body of evidence. | Section 2.1 (no formal framework applied) |
| **RESULTS** | | | |
| Study selection | **16a** | Describe the results of the search and selection, ideally with a flow diagram. | Section 3 + Figure 2 (PRISMA flow diagram) |
| Study selection | **16b** | Cite studies that appeared to meet criteria but were excluded, and why. | Section 3 and Figure 2 report the reports excluded at full-text assessment, grouped by reason (theoretical review, predictive without feedback, application without validation, and insufficient technical information); a per-report reference list of exclusions was not compiled. |
| Study characteristics | **17** | Cite each included study and present its characteristics. | Appendix A and Appendix B |
| Risk of bias in studies | **18** | Present assessments of risk of bias for each included study. | *No per-study scored RoB; study-level quality dimensions (baseline, duration, setting, attribution) reported in Appendices A/B and Sections 3.1–3.4; limitation stated in Section 4.* |
| Results of individual studies | **19** | Present, for each study, summary statistics and/or an effect estimate. | Appendices A/B + Figures 4–8 |
| Results of syntheses | **20a** | Summarise the characteristics and risk of bias of contributing studies. | Sections 3.1–3.4 (contributing-study quality dimensions—baseline, duration, setting, attribution—incorporated into the narrative synthesis; no formal scored RoB; see Section 2.1 and Section 4). |
| Results of syntheses | **20b** | Present results of all statistical syntheses conducted. | Sections 3.1–3.4 (descriptive synthesis) |
| Results of syntheses | **20c** | Present results of investigations of possible causes of heterogeneity. | Sections 3.1–3.4 |
| Results of syntheses | **20d** | Present results of all sensitivity analyses. | Not applicable (none performed) |
| Reporting biases | **21** | Present assessments of risk of bias due to missing results. | Section 2.1 / Section 4 (acknowledged as a limitation) |
| Certainty of evidence | **22** | Present assessments of certainty in the body of evidence. | Section 2.1 / Section 4 (not formally rated) |
| **DISCUSSION** | | | |
| Discussion | **23a** | Provide a general interpretation of the results. | Section 4 (Conclusions) |
| Discussion | **23b** | Discuss any limitations of the evidence included in the review. | Section 4 (Limitations) |
| Discussion | **23c** | Discuss any limitations of the review processes used. | Section 4 (Limitations: single database, single reviewer, no registration/RoB) |
| Discussion | **23d** | Discuss implications for practice, policy, and future research. | Section 4 |
| **OTHER INFORMATION** | | | |
| Registration and protocol | **24a** | Provide registration information, or state that the review was not registered. | Section 2.1 (the review was not registered) |

| | | | |
|---|---|---|---|
| Registration and protocol | **24b** | Indicate where the protocol can be accessed, or state a protocol was not prepared. | Section 2.1 (no formal protocol was prepared) |
| Registration and protocol | **24c** | Describe and explain any amendments to registration or protocol. | Not applicable |
| Support | **25** | Describe sources of financial or non-financial support and the role of funders. | Back matter (no external funding) |
| Competing interests | **26** | Declare any competing interests of review authors. | Back matter (Conflicts of Interest) |
| Availability of data, code and other materials | **27** | Report which materials are publicly available and where. | Appendices A/B + Data Availability Statement |

Reference: Page MJ, McKenzie JE, Bossuyt PM, et al. The PRISMA 2020 statement: an updated guideline for reporting systematic reviews. BMJ 2021;372:n71. doi:10.1136/bmj.n71
