# Supplementary material for: Intelligent Systems for Space Conditioning and Shading in Buildings: A Systematic Review of Artificial Intelligence Techniques and Their Energy Impact
Source: Sensors (Basel). 2026 Oct 10;26(20):6405. doi: 10.3390/s26206405 (PMC13708935; doi:10.3390/s26206405)
Supplement: Supplementary file 1 [file sensors-26-06405-s001.zip › sensors-4500105_Supplementary_Materials.pdf]

Supplementary Materials

Intelligent Systems for Space Conditioning and Shading in Buildings: A Systematic Review of Artificial Intelligence Techniques and Their Energy Impact

Manuscript ID: sensors-4500105

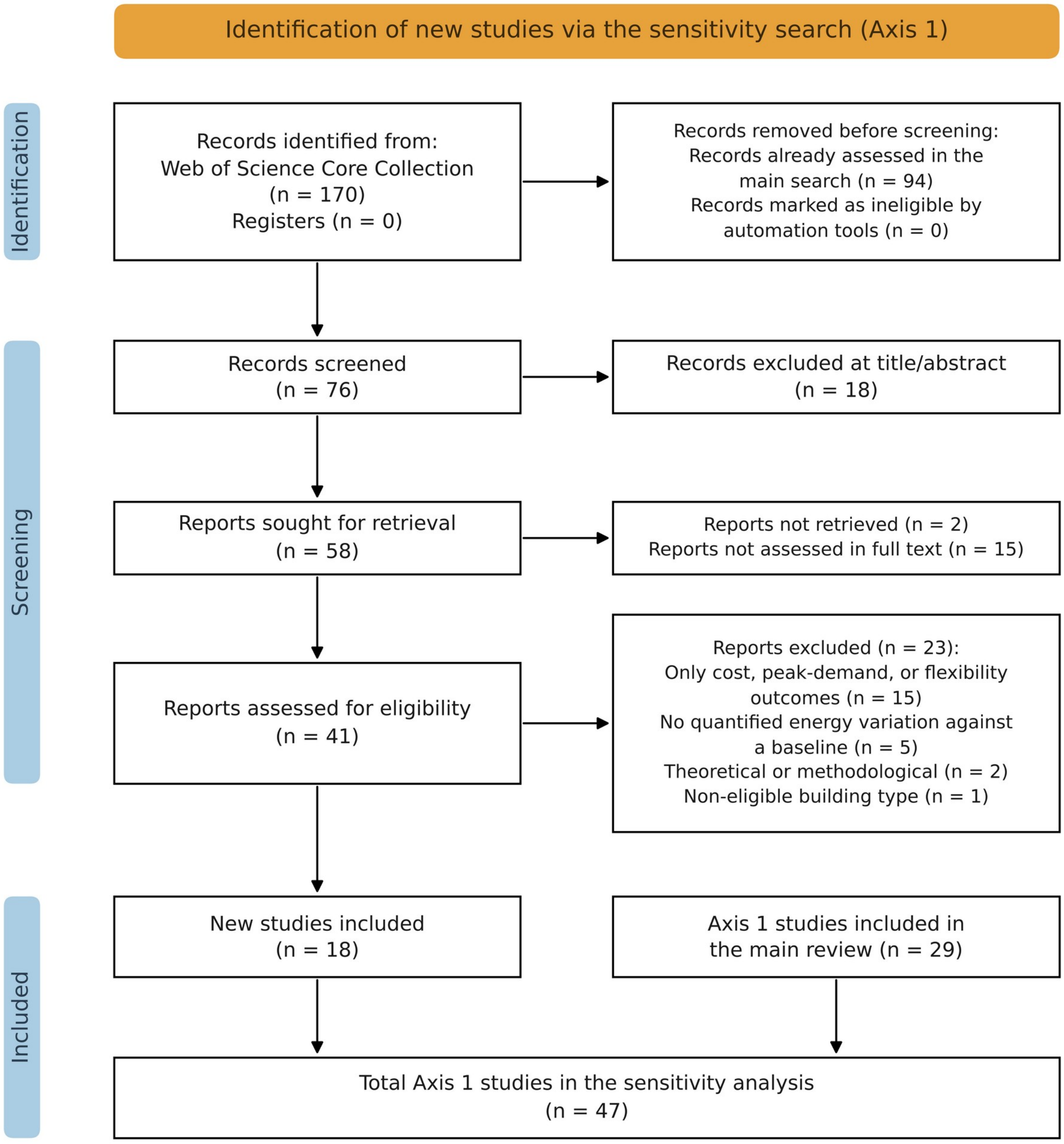

Figure S1. PRISMA 2020 flow diagram of the sensitivity search for Axis 1 (Web of Science Core Collection, 16 September 2026), which added the terms reinforcement learning, deep reinforcement learning, and model predictive control to the Axis 1 query. Records already assessed in the main search were removed before screening; the 18 new studies were not integrated into the main analysis and were used only to assess the robustness of its findings (Section 3.5).

Table S1. Studies included in the sensitivity analysis (Axis 1). Reference numbers correspond to the reference list of the main article.

Ref.	Study	Building	AI technique	Setting	Climate (Köppen)	Reported reduction (%)	Baseline
83	Drgoňa et al. (2020)	Office	White-box MPC	Real (79 days, transition seasons)	Cfb	53.5	Rule-based control
84	Joe & Karava (2019)	Office	Data-driven MPC	Real (heating and cooling test periods)	Dfa	16 (heating)	Feedback control
85	Dhaliwal et al. (2026)	Residential	Data-driven MPC	Simulation (EnergyPlus Python API, Ottawa 2019 AMY)	Dfb	4.7–7.4 (median 5.9)	Reactive thermostat control (1-h pre-conditioning)
86	Guo et al. (2025)	Office	DRL	Simulation (lab-calibrated hybrid simulator)	Dwa	21.4	Rule-based control
87	Heidari et al. (2022)	Residential	DQN	Simulation	Cfb	33.5	Rule-based control
88	Jiang et al. (2017)	Office	SVC/SVR (agent-based)	Simulation (field data)	Cfb	10.0	Conventional control
89	Li et al. (2015)	Office	MPC	Simulation (1 heating month)	Dfa	34.5	Conventional control
90	Liu et al. (2025)	Office	MPC with preference learning	Simulation (Python–EnergyPlus)	Dwa	44–66 (median 56.5)	Baseline control
91	Michailidis et al. (2018)	Office	Cooperative self-trained agents	Real (5–6 days, Nov. 2016)	Cfb	34.7	Rule-based control
92	Quang & Doan (2025)	Commercial	DRL (SAC) with online transfer learning	Simulation	Cfb	18	Rule-based control
93	Reynolds et al. (2018)	Office	ANN + GA + MPC	Simulation (February test week)	Cfb	25	Baseline heating schedule
94	Ebrahimi Saryazdi et al. (2026)	Residential	MPC	Simulation	BWh	7.1–16.1 (median 14.3)	On/off control
95	Tang et al. (2025)	Office	Multi-agent DRL (MADDPG)	Simulation (EnergyPlus–Python)	Dfa	24.1 (heating); 8.9 (cooling); median 16.5	Rule-based control
96	Tariq et al. (2025)	Office / residential	Multi-agent distributed RL (D-MAPPO)	Simulation (TRNSYS)	n.r.	23	D-MAPPO with default energy weights
97	Turley et al. (2020)	Residential	Occupancy prediction (ML) + MPC	Simulation (measured occupancy data)	BSk	5	Conventional schedule
98	Wang et al. (2024)	Office	DRL	Simulation (EnergyPlus)	Cfa	37	Existing HVAC schedule
99	Yang & Jradi (2025)	Office	Multi-objective MPC	Simulation (calibrated model)	Cfb	28.0	Existing control
100	Boutahri & Tilioua (2025)	Residential	Q-learning (RL)	Real (2-day validation; BOPTEST simulation)	Csa	8.8	Rule-based control

Reported reduction: energy-demand reduction reported by each study relative to its own baseline (total HVAC demand unless otherwise indicated); in the sensitivity analysis each study contributes one value (Section 3.5); where a study reports several scenarios, the range and the study median are given. n.r., simulation location not reported. DRL, deep reinforcement learning; DQN, deep Q-network; MPC, model predictive control; ANN, artificial neural network; GA, genetic algorithm; SVC/SVR, support vector classification/regression; SAC, soft actor–critic; MADDPG, multi-agent deep deterministic policy gradient; D-MAPPO, distributed multi-agent proximal policy optimization; AMY, actual meteorological year.
